# Microchiral pinwheel arrays based on achiral molecules

**DOI:** 10.1038/s41467-026-76089-z

**Published:** 2026-08-05

**Authors:** Jeong Yeon Han, Won Kyung Park, Byeongil Noh, Fumito Araoka, Sungwook Jeong, Byung Hak Jhun, Youngmin You, Yoonsu Park, Kyung Jin Lee, Jung-Moo Heo, Dong Ki Yoon

**Affiliations:** 1 Department of Chemistry, Korea Advanced Institute of Science and Technology (KAIST), Daejeon, Republic of Korea; 2 Department of Chemical Engineering and Applied Chemistry, Chungnam National University, Daejeon, Republic of Korea; 3 RIKEN Center for Emergent Matter Science, 2-1 Hirosawa, Wako, Saitama, Japan; 4 Department of Chemical and Biomolecular Engineering, Yonsei University, Seoul, Republic of Korea; 5 GIST InnoCORE AI-Nano Convergence Institute for Early Detection of Neurodegenerative Diseases, Gwangju Institute of Science and Technology, Gwangju, Republic of Korea; 6 Department of Molecular Science and Technology, Ajou University, Suwon, Republic of Korea

**Keywords:** Liquid crystals, Self-assembly, Self-assembly

## Abstract

From a molecular perspective, chirality is manifested in the intrinsic asymmetry of atomic arrangements that cannot be superimposed on their mirror images. Achiral molecules at nanometer scale can be assembled to have micron-scale chiral structures using physical stimuli, but the resulting chiral structures are often spatially limited, difficult to control over large areas, or unstable once the stimulus is removed. Here we show that a confined anisotropic molecular medium, derived from the nematic mesophase, undergoes a field-driven symmetry-breaking transition that generates micron-scale chiral structures. This approach provides a simple and scalable route to generate macroscopic chiral optical materials from molecularly symmetric precursors.

## Abstract (web summary)

Achiral molecules at nanometer scale can be assembled to have microscale chiral structures using physical stimuli, but achieving stable assemblies remains challenging. Here the authors demonstrate a field-driven symmetry-breaking transition of an anisotropic nematic mesophase that generates micron-scale chiral structures.

## Introduction

**Fig. 1:**
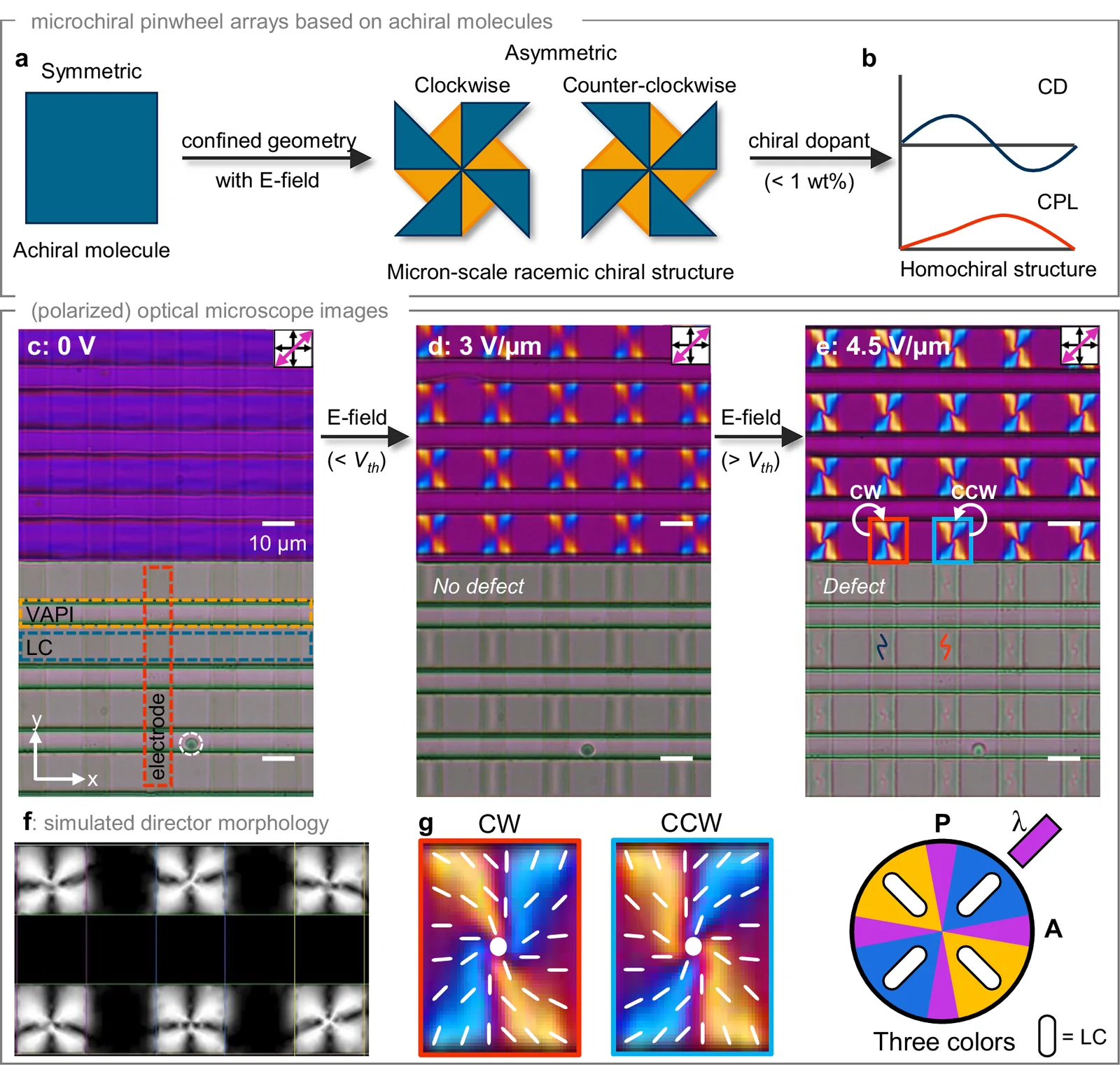
Formation of microchiral pinwheel arrays under confined geometry and electric field. **a** Schematic illustration showing the symmetry-breaking process of an achiral molecule confined within a microchannel under an applied electric field. The initially symmetric configuration evolves into asymmetric clockwise (CW) and counterclockwise (CCW) domains, forming micron-scale racemic chiral structures. **b** Conceptual diagram showing that the introduction of a small amount of a chiral dopant selects the handedness of the structure, leading to homochiral domains exhibiting macroscopic circular dichroism (CD) and circularly polarized luminescence (CPL). **c–e** POM images under different applied voltages. **c** At 0 V, the molecule shows uniform alignment without any chiral deformation. **d** At 3 V/μm (${V}_{F}$), no defect or symmetry breaking occurs. **e** At 4.5 V/μm (${V}_{T}$), CW and CCW spiral defects emerge alternately, forming a racemic array of chiral domains. The white circle marks the same position across images. Scale bars, 10 μm. **f** Simulated director field reproducing the alternating chiral domain pattern observed experimentally. **g** Visualization of the director orientation and optical response of CW and CCW pinwheel domains under polarized light (λ: full-wave plate, P: polarizer, A: analyzer). Each domain exhibits a distinct three-color birefringent texture corresponding to its molecular director.

Chirality is a fundamental structural motif in nature, spanning scales from molecules to macroscopic systems^1–4^. In biology, for example, proteins adopt right-handed α-helices from L-amino acids^5,6^. In materials science, however, chirality does not need to be built into molecules themselves. Even symmetric components can become left- or right-handed when symmetry is broken by geometry or external forces. A simple analogy is folding a symmetric sheet of paper into a pinwheel that rotates clockwise (CW) or counterclockwise (CCW), demonstrating how symmetry breaking can generate mirror-asymmetric shapes (Fig. 1a). Inspired by this idea, many studies have shown that chirality can be induced from achiral systems using physical stimuli such as mechanical motion^7,8^, hydrodynamic flow^9,10^, light^11–14^, or electric fields^15–18^. These works clearly demonstrate that chirality can be generated without molecular asymmetry. However, they also expose a central challenge, showing that the resulting chiral structures are often spatially limited, difficult to control over large areas, or unstable once the stimulus is removed. As a consequence, most practical chiral optical materials still rely on intrinsically chiral molecules or high dopant concentrations, which complicate synthesis, limit scalability, and hinder deterministic control of handedness at macroscopic scales^19,20^.

Here, we show that a confined anisotropic molecular medium, derived from the nematic mesophase, undergoes a field-driven symmetry-breaking transition that generates micron-scale chiral structures. Under geometric confinement and an in-plane electric field, the orientational field twists into randomly distributed CW and CCW spiral domains (Fig. 1a). Introducing a chiral dopant biases this symmetry breaking, converting the racemic domain distribution into a homochiral configuration and producing deterministic homochiral spiral defect scaffolds over large areas and open space (Fig. 1b). These field-maintained chiral configurations are then permanently encoded through chemical vapor deposition polymerization (CVP), yielding chiral polymer frameworks after removal of the molecular medium. The resulting chiral polymer scaffold acts as a template that transfers its helical environment to achiral luminophores, demonstrating that controlled chirality can be engineered through physical organization rather than molecular asymmetry. By combining low dopant loading, scalable processing, and solvent-free polymer fixation, this strategy provides a practical route to macroscopic chiral photonic materials based on achiral mesophase and polymer-forming components.

## Results

Field-programmed symmetry breaking in a mesophase

The anisotropic molecular medium, MLC-6608, is confined within vertically anchored polyimide (VAPI) microchannels prepared by PDMS molding, which impose strong homeotropic anchoring and align the y-axis (Supplementary Fig. 1)^21^. An interdigitated indium tin oxide (ITO) electrode array, oriented along the y-axis, generated an in-plane electric field along the x-axis, introducing an orthogonal stimulus that perturbs the collective molecular orientation (Supplementary Fig. 1)^22^.

Without an applied field, the VAPI boundaries and the molecule–air interface stabilize a uniform homeotropic arrangement (Fig. 1c)^23^. This state yields a spatially invariant magenta texture under a polarized optical microscope (POM), confirming z-axis alignment across the entire channel. Full-wave-plate (λ-plate) imaging reveals weak, localized birefringence near the sidewalls, reflecting slight deviations in local orientation imposed by surface anchoring (Supplementary Fig. 2).

Applying a 3 V μm^–1^ AC field (10 kHz, threshold of Freedericksz transition, ${V}_{F}$) uniformly tilts the molecular director, describing how the anisotropic medium is oriented in space, without breaking mirror symmetry or forming chiral defects (Fig. 1d). Above the threshold for chiral twist formation (4.5 V μm^–1^ at 10 kHz, ${V}_{T}$), the in-plane electric torque overwhelms vertical anchoring, leading to localized director instabilities that evolve into chiral twist–spiral defects (Fig. 1e). This pinwheel texture is better described as a three-dimensional director distortion than as a simple two-dimensional −1 point defect (Supplementary Fig. 3)^24^. It arises from the combined effects of vertical anchoring, sidewall confinement within the VAPI microchannels, and the in-plane electric field. Because MLC–6608 has negative dielectric anisotropy (Supplementary Table 1), the director tends to reorient perpendicular to the applied field while simultaneously satisfying the anchoring conditions imposed by the VAPI boundaries and the molecule–air interface. This competition promotes coupled splay, bend, and twist deformations, yielding the pinwheel-like twist–spiral arrangement. Under a λ-plate, each chiral domain displays a distinct three-color birefringent pattern (Fig. 1g), enabling direct visualization of the twist sense and confirming the formation of racemic supramolecular chirality in the pixel^25^. Bright-field images show that defects nucleate predominantly at electrode gaps, where the balance between anchoring elasticity and in-plane electric torque is most competitive (Fig. 1d, e). Simulations reproduce the radial distortion of the director field (Fig. 1f), consistent with a continuum elastic model that lacks stochastic fluctuations—explaining why the experiment yields both CW and CCW domains.

### Formation of chiral nanofibers via CVP

**Fig. 2:**
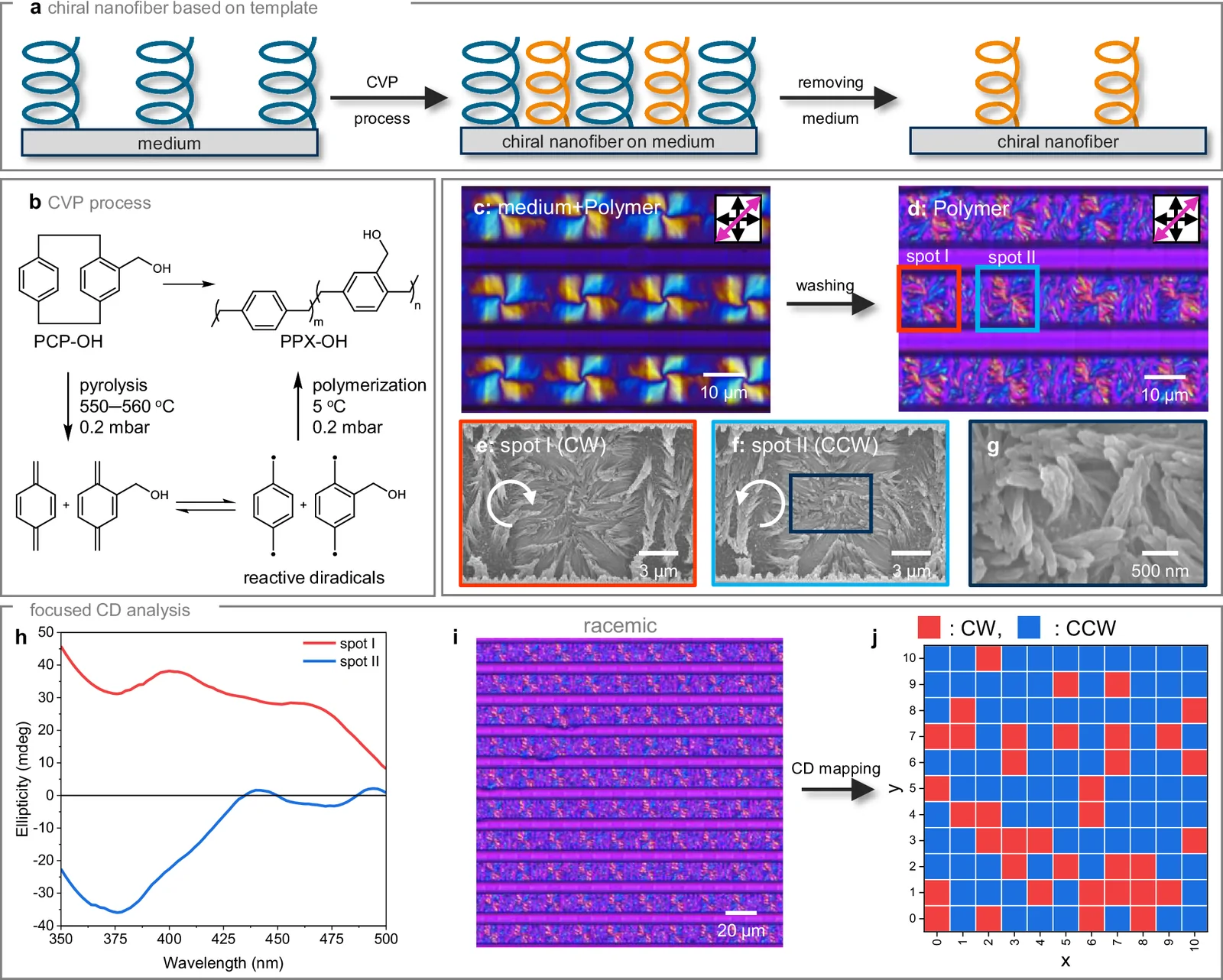
Formation and characterization of chiral polymer nanofibers. **a** Schematic illustration of the fabrication process. 4-hydroxymethyl[2.2]paracyclophane (PCP–OH) was polymerized via chemical vapor deposition polymerization (CVP) to yield chiral polymer nanofibers after medium removal. **b** CVP mechanism: PCP–OH dimers were pyrolyzed at 550–560 °C under 0.2 mbar to generate reactive diradical intermediates, which polymerized at 5 °C to form poly(hydroxymethyl-p-xylylene) (PPX–OH). **c**, **d** Polarized optical microscopy (POM) images of the medium–polymer composite immediately after CVP (**c**) and the resulting polymer after washing out the medium (**d**), showing preserved chiral textures. **e**, **f** Scanning electron microscopy (SEM) images at spots I and II in (**d**) corresponding to clockwise (CW) and counterclockwise (CCW) domains, respectively. **g** High-magnification SEM image revealing twisted nanofiber (CCW) morphology. **h** Focused circular dichroism (CD) spectra recorded at spots I (red, CW) and II (blue, CCW), showing mirror-image Cotton effects. **i**, **j** CD mapping analysis of the racemic polymer film: **i** representative POM image of the measured region; **j** corresponding spatial chirality map showing randomly distributed CW (red) and CCW (blue) domains. Scale bars: 10 µm (**c**, **d**), 3 µm (**e**, **f**), 500 nm, (**g**) 20 µm (**i**).

The field-generated chiral templates are encoded into permanent polymer architectures using CVP of the achiral PCP–OH (4-hydroxymethyl[2.2]paracyclophane) monomer, deposited directly onto the orientationally ordered mesophase medium (Fig. 2a, b). PCP–OH was selected because [2.2]paracyclophane derivatives enable solvent-free polymerization, allowing the preorganized director field to be fixed without solvent-induced disruption or phase separation. The resulting poly(hydroxymethyl-p-xylylene) (PPX–OH) scaffold preserves the field-induced director morphology after removal of the mesophase, providing a robust model system for mesophase-to-polymer chirality transfer. During CVP, PCP–OH undergoes pyrolysis at 550–560 °C under 0.2 mbar to generate reactive p-quinodimethane diradicals, which subsequently condense and polymerize at 5 °C to form PPX–OH nanofibers preferentially aligned along the local director of the underlying medium (Supplementary Fig. 4)^26–29^.

POM images obtained after the CVP process show that the characteristic chiral texture is maintained after polymer deposition (Fig. 2c). After removing the mesophase medium by solvent washing, the optical pattern remains unchanged (Fig. 2d), confirming that the symmetry-broken orientational configuration has been permanently fixed by CVP-directed polymer growth. The formation of PPX–OH nanofibers is further confirmed by infrared (IR) and X-ray photoelectron spectroscopy (XPS) analyses (Supplementary Fig. 5). The IR spectrum after CVP shows the appearance of a characteristic broad O–H stretching band near 3300 cm^−1^, consistent with the formation of PPX–OH. XPS analysis reveals a distinct O 1 *s* peak (~532 eV). After washing, the disappearance of the N 1 *s* peak (~399 eV) revealed the complete removal of residual mesophase molecules, leaving a pure polymer framework. Scanning electron microscopy (SEM) of representative domains (spots I and II) reveals well-defined CW and CCW spiral helices, and higher-magnification images confirm twisted assemblies of PPX–OH nanofibers inherited from the spiral defects (Fig. 2e–g). These results demonstrate that the field-induced mesophase chirality is translated into a mesoscale chiral polymer architecture composed of achiral PPX–OH nanofibers, rather than molecular-level chirality of the polymer backbone. Focused circular dichroism (CD) spectroscopy (Fig. 2h) shows opposite-sign Cotton effects from representative CW and CCW regions. Because these spectra were collected from different microscopic domains in a racemic film, local variations in polymer thickness, fiber density, orientation, and scattering can produce deviations from ideal mirror-image symmetry. Spatial CD mapping (Fig. 2i, j) reveals a racemic distribution of chiral domains, illustrating the formation of a macroscopic chiral polymer framework through confinement and the applied field.

### Homochiral induction assisted by chiral doping

**Fig. 3:**
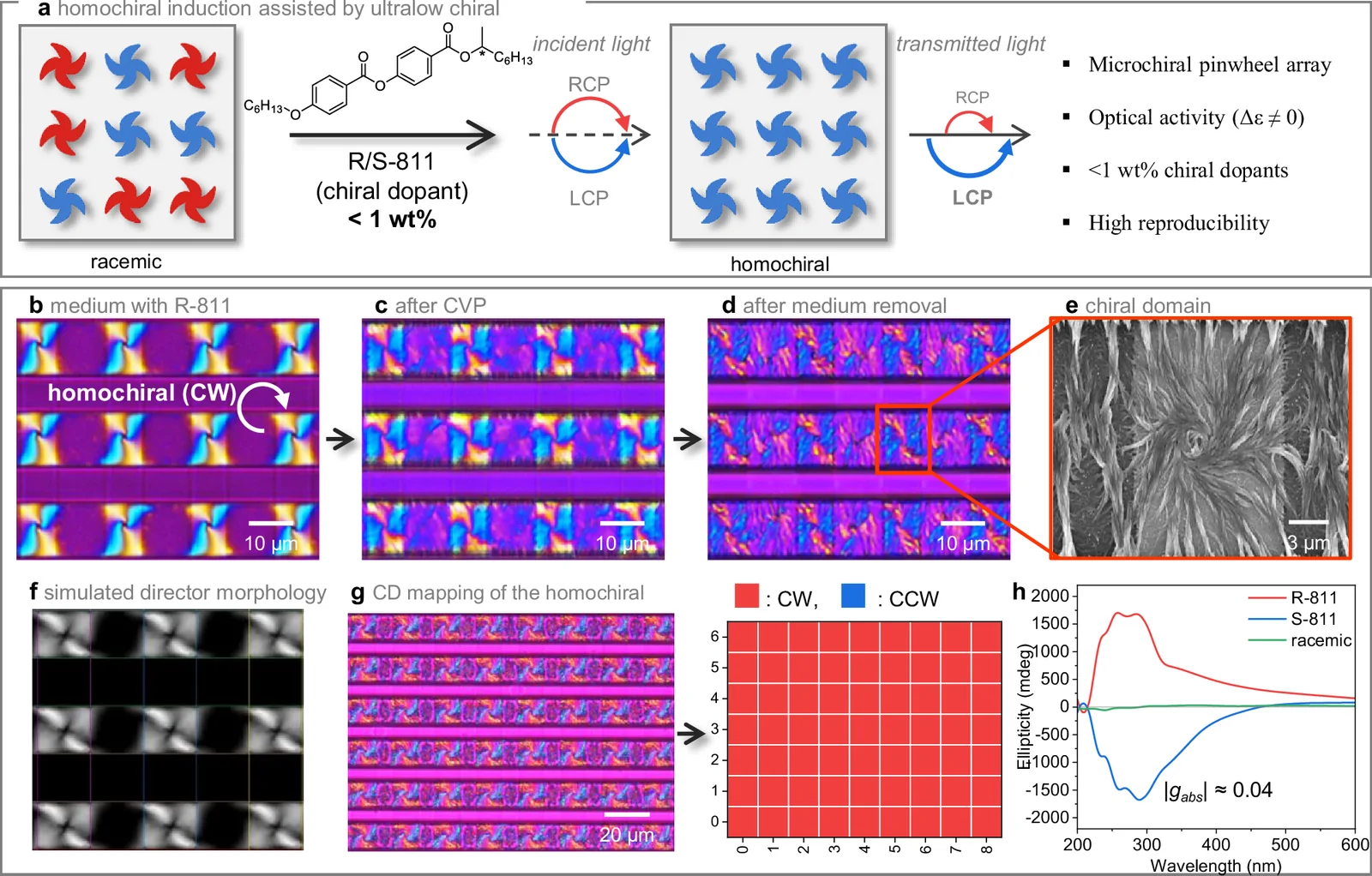
Homochiral induction by a chiral dopant. **a** Schematic illustration showing the transition from a racemic to a homochiral pinwheel array by introducing a small amount (<1 wt%) of chiral dopant. The induced homochiral structure exhibits optical activity (Δε ≠ 0) and strong circular selectivity toward right- (RCP) or left-circularly polarized (LCP) light. **b–d** Polarized optical microscopy (POM) images of (**b**) the doped medium with R-811 showing uniform clockwise (CW) domains, **c** after chemical vapor deposition polymerization (CVP) of 4-hydroxymethyl[2,2]paracyclophane (PCP–OH), and **d** after medium removal, where the single-handed structure remains intact. **e** Scanning electron microscopy (SEM) image of a representative CW domain, confirming the preserved spiral morphology after medium removal. **f** Simulated liquid crystal (LC) morphology showing unidirectional director rotation consistent with homochiral alignment. **g** Focused circular dichroism (CD) mapping of the polymer scaffold, revealing complete CW homochirality across the measured area. **h** CD spectra of R-811–, S-811–doped, and undoped (racemic) samples. The doped systems exhibit strong, opposite-signed Cotton effects (Δε ≠ 0), with R-811 yielding positive and S-811 negative signals, confirming enantiomer-dependent homochirality. Scale bars: 10 µm (**b–d**), 3 µm (**e**), 20 µm (**g**).

The field-induced chiral domains described above form a racemic array of CW and CCW spirals, yielding no net optical activity. Achieving large-area homochirality in such achiral systems has remained challenging, since spontaneous symmetry breaking typically produces equal populations of each-handed domains. To impose deterministic handedness, we introduced a chiral dopant into the mesophase medium and examined its concentration-dependent effect on pinwheel formation under the threshold field for chiral-twist formation (4.5 V µm^–1^, 10 kHz, (${V}_{T}$)). At low R/S-811 loadings, the field-induced pinwheel arrays showed incomplete handedness selection, with minor oppositely handed domains coexisting with the dominant handedness (Supplementary Fig. 6), indicating mixed, non-racemic arrays. At 0.7 wt%, uniform single-handed arrays were reproducibly obtained: R-811 generated homochiral CW spirals, whereas S-811 generated homochiral CCW spirals (Fig. 3b, Supplementary Fig. 6). At higher dopant loading, fingerprint-like cholesteric textures dominated and well-defined pinwheels no longer formed, indicating that excessive loading promotes intrinsic cholesteric ordering rather than field-induced pinwheel formation (Supplementary Fig. 6). Deterministic homochiral pinwheel formation therefore requires an optimized concentration window in which the dopant biases the handedness of the field-induced symmetry breaking without suppressing the confinement-driven morphology. After CVP and subsequent removal of the mesophase medium, including MLC–6608 and the chiral dopant, both polymer scaffolds preserve their single-handed morphologies (Fig. 3c–e for R-811, Supplementary Fig. 7 for S-811). Simulations (Fig. 3f) reproduce the unidirectional director rotation, consistent with dopant-induced helical selectivity. Focused CD mapping further confirms the formation of distinct CW and CCW domains corresponding to each dopant (Fig. 3g, Supplementary Fig. 8, respectively).

The optical response reflects this structural asymmetry. CD spectroscopy of the undoped array shows a flat baseline, confirming racemic symmetry in macroscopic field of view, whereas doped samples exhibit strong, single-sign Cotton effects—positive for R-811 and negative for S-811 (Fig. 3h). The CD amplitude exceeds 1500 mdeg, corresponding to a $\left|{g}_{{abs}}\right|$ ≈ 0.04, and reverses sign with the dopant. We next performed control experiments to evaluate possible solid-state optical artifacts. CD spectra measured at sample-rotation angles of 0°, 45°, 90°, and 135° and in front- and back-side geometries retained the same handedness-dependent sign and spectral shape (Supplementary Fig. 9), indicating that the response is not dominated by linear dichroism, linear birefringence, or sample-orientation effects. We further varied the PCP–OH precursor amount during CVP to control the polymer scaffold thickness (Supplementary Fig. 10). The absolute CD intensity and (${g}_{{abs}}$) values varied with precursor amount, with the 30 mg condition giving the strongest response, indicating that the chiroptical magnitude is influenced by scaffold thickness and/or nanofiber density. Nevertheless, all samples retained the same CD sign and similar Cotton-effect profiles, supporting that the handedness-dependent CD response originates from the chiral nanofiber architecture, while its magnitude is modulated by the deposited scaffold amount.

The applicability of this induction mechanism was further examined using chiral dopants with distinct stereochemical origins and different helical twisting powers. R/S-5011, possessing axial chirality, and CB15, exhibiting point chirality, produced uniform homochiral domains at optimized concentrations of 0.08 wt% and 1.1 wt%, respectively, under the same field condition (Supplementary Figs. 11–13). The lower concentration required for R/S-5011 reflects its stronger helical twisting power in MLC–6608. Notably, although R/S-5011 was used at a much lower concentration than R/S-811, both dopant systems produced a comparable cholesteric pitch of approximately 13 μm under their optimized conditions (Supplementary Fig. 14), indicating that different dopant concentrations are required to provide comparable chiral bias for reproducible handedness selection. POM, SEM, and CD analyses consistently confirm that the induced handedness follows the dopant enantiomer.

Importantly, these results highlight the remarkable efficiency of this field-assisted confinement strategy. Conventional cholesteric, ferroelectric liquid crystals or polymeric platforms rely on heavy dopant loadings, often exceeding 20–30 wt%, to achieve macroscopic homochirality^30–32^. In contrast, our system generates thoroughly homochiral architectures with <1 wt% dopant, two orders of magnitude lower than previously reported. This enhancement arises from the cooperative coupling between the in-plane electric field and vertical confinement, which amplifies molecular torque into macroscopic alignment. The underlying mechanism can be rationalized using the elastic free-energy model of chiral nematic systems (see Supplementary Note 1 for theoretical details).

### Circularly polarized luminescence from dye-coated homochiral templates

**Fig. 4:**
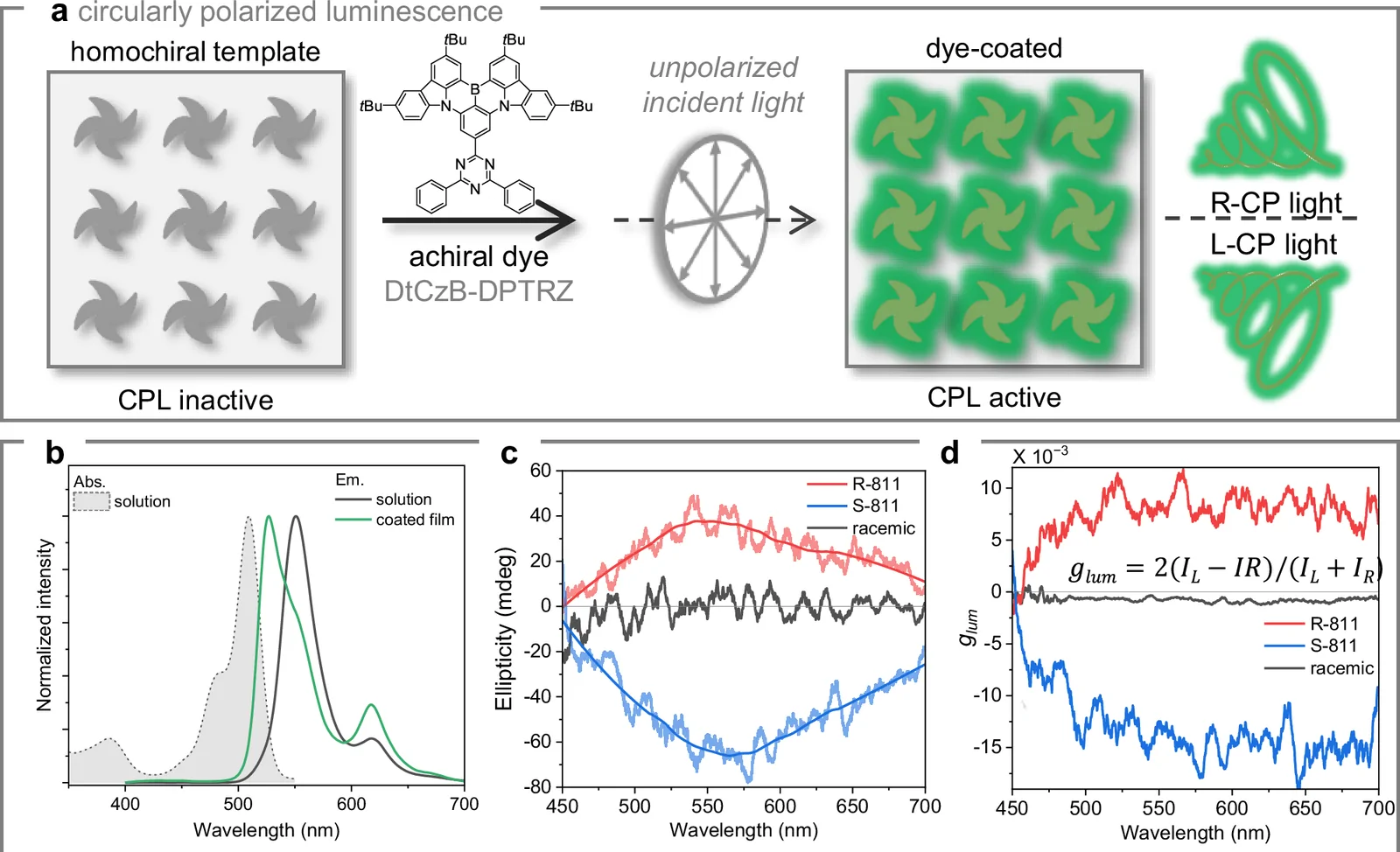
CPL from dye-coated homochiral polymer scaffolds. **a** Schematic illustration showing that the homochiral polymer scaffold obtained via chemical vapor deposition polymerization (CVP) is intrinsically circularly polarized luminescence (CPL) inactive, but becomes CPL active upon spin-coating with an achiral emissive dye. **b** Absorbance spectra of dye solution in chlorobenzene with 1 × 10^–5^ M and photoluminescence (PL) spectra of dye solution with the same concentration and coated film under 365 nm excitation. **c**, **d** CPL spectra of dye-coated films prepared on R-811 and S-811 templated scaffolds exhibit mirror-image ellipticity with $\left|{g}_{{lum}}\right|$ ≈ 8.3 × 10^–3^ and 14.4 × 10^–3^ from achiral dyes on homochiral polymer scaffolds.

To demonstrate the optical functionality of the homochiral resultant, an achiral green-emitting thermally activated delayed fluorescence (TADF) dye, DtCzB-DPTRZ, is spin-coated onto the chiral nanofiber surface to produce right-handed and left-handed circularly polarized luminescence (R-CPL and L-CPL, respectively) emissions from the corresponding homochiral templates (Fig. 4a). The dye exhibits an absorption maximum at 509 nm and photoluminescence centered at 551 nm in solution (Fig. 4b). The emission spectrum of the coated film closely matches that of the dye in solution, confirming incorporation of the dye into the chiral polymer matrix (Fig. 4b, c).

Under UV excitation, the coated films exhibit clear mirror-image CPL that directly followed the handedness of the underlying scaffold—positive for CW (R-CPL) and negative for CCW (L-CPL) configurations—with dissymmetry factors of $\left|{g}_{{lum}}\right|$ ≈ 8.3 × 10^–3^ and 14.4 × 10^–3^, respectively (Fig. 4d). To evaluate possible sample-orientation artifacts in the CPL measurements, we recorded CPL spectra during full in-plane sample rotation in 45° steps from 0° to 315° (Supplementary Fig. 15). The R-811-templated scaffold retained positive CPL at all rotation angles, whereas the S-811-templated scaffold retained negative CPL. Although the ellipticity and (${g}_{{lum}}$) values showed moderate angular variation, the CPL sign and overall spectral shape were preserved for each handed scaffold, supporting that the observed CPL is not dominated by sample-orientation artifacts. In addition, the scaffold CD band lies mainly in the ultraviolet region, whereas the dye emission occurs around 540–550 nm, and the estimated selective-reflection wavelength is far outside the visible emission region. These controls support that the CPL arises from chirality transfer from the homochiral PPX–OH scaffold to the achiral luminophore, rather than being dominated by sample orientation, CD leakage, or selective-reflection effects.

These results demonstrate that the achiral luminophore inherits the helically polarized optical signal of the polymer scaffold, generating chiral emission through physical templating rather than molecular modification. To further demonstrate the applicability of this templating strategy, we apply the same homochiral scaffold to an achiral organometallic phosphor, PtN, which emits in the red region. Spin-coated PtN films displayed red phosphorescence with a peak wavelength at approximately 560 nm and again produced mirror-image CPL signals with $\left|{g}_{{lum}}\right|$ values on the order of (2–3) × 10^–2^, depending on the template handedness (Supplementary Fig. 16).

Beyond light emission, the homochiral polymer framework can provide a versatile platform for diverse chiroptical and functional applications, including chiral sensing, polarization-controlled displays, and optical information encryption, where stable and scalable chirality transfer from achiral components is highly desirable^33–36^. The ability to induce chirality under such low dopant loading simplifies material design while minimizing structural perturbation, offering a robust and scalable route to programmable chiral scaffolds based on soft materials.

## Discussion

In this work, we addressed the long-standing challenge of realizing large-area chirality from intrinsically achiral systems. Our results establish a scalable strategy to generate macroscopic chirality without molecular asymmetry or elaborate synthesis. By coupling electric-field–induced director distortion with geometric confinement, achiral molecules spontaneously evolve into spiral templates. Introducing only trace amounts of chiral dopant directs the handedness of these structures, producing uniform homochiral frameworks that remain stable after polymer fixation through CVP. The resulting polymer scaffolds effectively transfer orientational order from the nanoscale to the macroscale, yielding optical chirality upon dye coating. Achiral luminophores deposited on these templates exhibit distinct circularly polarized luminescence, demonstrating that optical handedness can be engineered through field-guided templating. This approach combines dopant loading, straightforward processing, and broad material compatibility. Beyond CPL generation, the demonstrated templating principle offers a versatile foundation for chiral sensing, polarization-selective displays, and optical data encryption, paving the way toward universal and scalable chiral photonic systems.

## Methods

### Materials

MLC6608 anisotropic material was obtained from Merck (lot no. 0000433542). Chiral dopants R-811 (≥99.5%) and S-811 (≥99.5%) were purchased from Chemfish Tokyo; R-5011 (>99.9%) from Merck; S-5011 (≥98.0%) from Daken Chemical Co., Ltd.; and CB15 (≥98.0%) from Tokyo Chemical Industry Co., Ltd. Vertical-alignment polyimide (VAPI) and planar-alignment polyimide (PAPI) were supplied by Shenzhen Dalton Electronic Materials Co., Ltd. The PDMS precursor (Sylgard 184B) was obtained from Dow Chemical and mixed with the curing agent at a 10:1 weight ratio. Trichloro(1H,1H,2H,2H-perfluorooctyl)silane (FOTS, 97%) was purchased from Sigma-Aldrich. 4-Hydroxymethyl[2.2]paracyclophane (PCP–OH) was synthesized and confirmed by 1H and 13 C NMR spectroscopy^29^ (Supplementary Figs. 17–19).

### PDMS mold preparation

A Si micro-channel with a 10 µm width and a 5 µm depth used as a master mold was sequentially rinsed with acetone, ethanol, and DI water. After cleaning, the O_2_ plasma was treated for 15 min to remove residues. Then, the cleaned Si micro-channel was placed in the vacuum pump with FOTS (20 µL) in the weighing dish and put under vacuum for 20 min. Fluorosilanized Si micro-channel was rinsed with ethanol again and heated at 120 °C for 1 h. PDMS mold was prepared using a PDMS precursor and curing agent in a 10:1 weight ratio. The mixture was poured onto the Si micro-channel, and air bubbles were removed by placing the poured mixture in the vacuum pump for 1 h. The air removed PDMS mixture was cured for 4 h in an oven at 65 °C. The cured flexible PDMS mold was detached from the Si master mold.

### VAPI channel substrate preparation

ITO patterned quartz substrates were sequentially rinsed with acetone, ethanol, and DI water. After cleaning, the O_2_ plasma was treated for 15 min to remove residues. 2.5 µL of VAPI was dropped on the substrate, and the PDMS mold was put on the substrate with the channel perpendicular to the ITO electrodes. PDMS molded substrate was soft-baked at 90 °C for 30 min and the PDMS mold was removed. Then, the substrate was hard-baked at 150 °C for 2 h. A schematic illustration of the process is shown in Supplementary Fig. 1.

### Chemical vapor deposition polymerization (CVP)

The LC template was mounted in a custom-made CVD polymerization chamber (TeraLeader, Republic of Korea). After achieving a base pressure of 2 × 10⁻^1^ mbar under argon flow (99.999%), 30 mg of PCP–OH dimer was used as the standard optimized condition for CVP unless otherwise stated. The PCP–OH dimer was sublimed at 90–120 °C and transferred to the pyrolysis zone, where it was thermally cleaved to p-quinodimethane diradical monomers at 550–560 °C. The reactive monomers were carried into the deposition chamber (80 °C) and polymerized at 5 °C on the mesophase medium. Total deposition time was 60 min. All steps were performed under continuous argon flow ( ~ 15 sccm) to prevent oxidation. For the CD thickness-dependence control experiments, the amount of PCP–OH dimer was varied from 20 to 60 mg while all other CVP conditions were kept identical. A schematic illustration of the process is shown in Supplementary Fig. 4^26^.

### LC and chiral dopant mixture preparation

For chirality-controlled arrays, chiral dopants were mixed with the achiral nematic host MLC6608 at various concentrations: 0.7 wt% for R/S-811, 0.08 wt% for R/S-5011, and 1.1 wt% for CB15. The mixtures were first dissolved in dichloromethane (DCM), followed by solvent evaporation under mild heating (40 °C) and magnetic stirring for 12 h to ensure complete homogenization. The resulting mixtures were stored in sealed vials at room temperature prior to use.

### Fabrication of chiral template

The LC mixture was filled into the VAPI microchannels via capillary action at room temperature. An alternating-current (AC) electric field was applied using a function generator (EDU33211A, Keysight) connected to a voltage amplifier (A400, FLC Electronics). Unless otherwise stated, the applied voltage was 45 V_p_ at 10 kHz frequency. Samples were observed in situ under a polarized optical microscope during the field application. The mesophase template consists of the negative-dielectric-anisotropy nematic host MLC–6608, with or without a chiral dopant, confined within the VAPI microchannel array and addressed by an in-plane electric field applied through the interdigitated ITO electrodes. PCP–OH is introduced only during the subsequent CVP step as a vapor-phase precursor, and MLC–6608 and the dopant are removed after polymerization to leave the PPX–OH scaffold.

### Medium removal after CVP

Following CVP, the substrates were immersed in ethanol for 10 min to remove surface residues, followed by immersion in n-hexane for 6 h to dissolve the medium. The samples were then air-dried for 1 h under a clean hood. No additional annealing was performed to avoid distortion of the polymer framework.

### Wedge cell preparation

Glass substrates (2.5 cm × 1.25 cm) were sequentially rinsed with acetone, ethanol, and DI water. After cleaning, the O_2_ plasma was treated for 15 min to remove residues. 20 µL of PAPI was spin-coated with 3500 rpm for 1 min on the substrate. Spin-coated substates were soft-baked at 90 °C and chiral dopant mixture preparation for 30 min and hard-baked at 200 °C for 2 h. To generate the nanogrooves on the substrates, they were treated using the rubbing process by a rubbing machine (RMS-50-M, Namil Optics). The substrates were sandwiched with Kapton tape with 100 µm thickness and UV-curable glue. The mesophase medium was injected into the sandwich cell.

### Optical characterization

Textures were observed using a polarized optical microscope (LV100POL, Nikon) equipped with a CCD camera (DS-Ri1, Nikon). Quarter-wave plates (λ = 530 nm) were used when indicated. Images were captured under crossed polarizers with a 50× objective. CD spectra were recorded using a J-1500 circular dichroism spectrophotometer (Jasco) at 25 °C in reflection mode. Each spectrum was averaged over three scans from 200–800 nm (1 nm step, 100 nm/min). Localized CD spectra were obtained using a micro-focused CD system (in collaboration with Dr. F. Araoka, RIKEN). A 10 µm beam spot was scanned over a 200 × 200 µm² area with a 10 µm step size, and ellipticity maps were reconstructed from integrated intensity. CPL spectra were collected using a CPL-300 spectrometer (Jasco) under 365 nm excitation. Emission was recorded from 400–700 nm with a step size of 1 nm and an integration time of 0.5 s per step. Dissymmetry factors (${g}_{{lum}}$) were calculated as ${g}_{{lum}}=2({I}_{L}-{I}_{R})/({I}_{L}+{I}_{R}).$

### Microscopy and structural analysis

Surface morphology and defect structures were observed using a field-emission SEM (SU8230, Hitachi). Samples were coated with a ~3 nm osmium layer (Neoc-Pro, Meiwafosis) to prevent charging. Images were acquired at 5.0 kV accelerating voltage, 10 µA probe current, and 5–10 mm working distance under high vacuum. Fourier-transform infrared spectra were obtained with a PerkinElmer Spectrum Two instrument using an ATR diamond crystal. Spectra were averaged over 64 scans with 4 cm^–^^1^ resolution in the range 4000–600 cm^–1^. The appearance of characteristic aromatic C = C and C–O–H stretching bands confirmed PPX–OH formation after CVP. XPS measurements were performed using a Thermo Scientific K-Alpha+ spectrometer equipped with a monochromatic Al Kα X-ray source (hν = 1486.6 eV) operated at 12 kV and 6 mA. The base pressure in the analysis chamber was maintained below 1 × 10^–9^ mbar during measurements. Survey spectra were collected over the 0–1200 eV range with a pass energy of 200 eV, and high-resolution scans for C 1 *s*, O 1 *s*, and N 1 *s* regions were acquired with a pass energy of 20 eV and step size of 0.1 eV. Charge compensation was achieved using a low-energy electron flood gun, and all binding energies were calibrated with respect to the C 1 *s* peak at 284.8 eV. The as-prepared template film exhibited distinct N 1 *s* peaks in MLC6608. After the washing process following CVP, the N 1 *s* signal completely disappeared, while the C 1 *s* and O 1 *s* peaks corresponding to the PPX–OH polymer backbone remained (Supplementary Fig. 4). This confirms that the medium and dopant components were entirely removed, leaving only the chiral polymer framework on the substrate. ^1^H and ^13^C NMR spectra were recorded on a 400 MHz spectrometer (Bruker Avance III HD) in CDCl_3_ at 25 °C. Chemical shifts were referenced to tetramethylsilane (TMS, 0 ppm). Detailed synthetic procedures and full NMR assignments are provided in the Supplementary Materials.

### Director simulation

Director field distributions and electric-field responses were simulated using TechWiz LCD 3D (Sanayi System Co., Ltd.). The elastic constants *K*_*1*_, *K*_*2*_, and *K*_*3*_ were set to 8.7, 3.7, and 10.0 pN, respectively, corresponding to MLC6608 parameters. Boundary conditions were set to homeotropic anchoring (W = 1 × 10–3 J m^–2^) on the VAPI walls and an in-plane electric field (E = 4.5 V µm^–1^). Calculated director fields were used to reproduce qualitative POM contrast patterns.

## Supplementary information

## Source data

## Data Availability

Source data are provided with this paper. All data generated in this study are provided in the main text, Supplementary Information and Source data file. All data are available from the corresponding author upon request. Source data are provided with this paper.
