# Supplementary material for: Microchiral pinwheel arrays based on achiral molecules
Source: Nat Commun. 2026 Aug 5;17:9413. doi: 10.1038/s41467-026-76089-z (PMC13538584; doi:10.1038/s41467-026-76089-z)
Supplement: Supplementary file 1 [file 41467_2026_76089_MOESM1_ESM.pdf]

Supplementary Information

Microchiral pinwheel arrays based on achiral molecules

Authors: Jeong Yeon Han[1], Won Kyung Park[2], Byeongil Noh[2], Fumito Araoka[3], Sungwook Jeong[1], Byung Hak Jhun[4], Youngmin You[4], Yoonsu Park[1], Kyung Jin Lee*[2], Jung-Moo Heo*[1,5,6], Dong Ki Yoon*[1,5]

Affiliations:

[1]Department of Chemistry, Korea Advanced Institute of Science and Technology (KAIST), Daejeon 34141, Republic of Korea

[2]Department of Chemical Engineering and Applied Chemistry, Chungnam National University, Daejeon 34134, Republic of Korea

[3]RIKEN Center for Emergent Matter Science2-1 Hirosawa, Wako, Saitama 351-0198, Japan.

[4]Department of Chemical and Biomolecular Engineering, Yonsei University, Seoul 03722, Republic of Korea

[5]GIST InnoCORE AI-Nano Convergence Institute for Early Detection of Neurodegenerative Diseases, Gwanjgu Institute of Science and Technology, 61005 Gwangju, Republic of Korea

[6]Department of Molecular Science and Technology, Ajou University, Suwon 16499, Republic of Korea

*Corresponding author. Email: kjlee@cnu.ac.kr, jungmoo@ajou.ac.kr, nandk@kaist.ac.kr

Table of Contents

1. Supplementary Note 1......p. 2
2. Table 1......p. 3
3. Figure S1~S17......p. 4
4. Synthetic Procedures......p. 21
5. Figure S18~S20......p. 21
6. References......p. 24

Supplementary Note 1. Theoretical description of chiral induction

When a chiral dopant is introduced into a nematic mesophase, liquid crystalline molecule, it imposes a preferred sense of twist on the director field. The elastic free energy of an achiral nematic mesophase can be expressed as

$$F_N = F_N = \frac{1}{2}K_1(\nabla \cdot \hat{n})^2 + \frac{1}{2}K_2(\hat{n} \cdot \nabla \times \hat{n})^2 + \frac{1}{2}K_3(\hat{n} \times \nabla \times \hat{n})^2 \qquad (1)$$

where K_1, K_2, and K_3 are the elastic constants for splay, twist, and bend deformations, respectively. In an achiral LC, there is no energetic distinction between left- and right-handed twists; hence, field-induced perturbations generate both clockwise (CW) and counterclockwise (CCW) distortions with equal probability. Upon addition of a chiral dopant, the twist term is modified as follows:

$$F_{CLC} = \frac{1}{2}K_1(\nabla \cdot \hat{n})^2 + \frac{1}{2}K_2(\hat{n} \cdot \nabla \times \hat{n} - \frac{2\pi}{p})^2 + \frac{1}{2}K_3(\hat{n} \times \nabla \times \hat{n})^2 \qquad (2)$$

where p denotes the cholesteric pitch, whose sign defines the handedness imposed by the dopant. In this case, the elastic energy becomes dependent on the twist sense, leading to preferential selection of one handedness during director relaxation. In our system, the chiral dopant does not directly generate the chiral structure but instead acts as a helicity selector, determining the twist direction of the field-induced distortion. As a result, large-area homochirality can be achieved with extremely low dopant concentrations (< 1 wt%), since the dopant merely biases the symmetry breaking rather than driving it. This mechanism is consistent with the observed defect evolution: in the absence of an applied field, the LC director remains predominantly along the z-axis with localized alignment near the channel walls (Supplementary Fig. 10). No cholesteric texture is observed within the channels because the intrinsic pitch (~13 µm in Supplementary Fig. 11) of the doped LC exceeds both the channel width and depth, and geometric confinement suppresses spontaneous helical formation.

Supplementary Table 1Dielectric and elastic parameters of MLC–6608.

Parameter	Symbol	Value
Parallel dielectric constant	$\varepsilon_{\parallel}$	3.6
Perpendicular dielectric constant	$\varepsilon_{\perp}$	7.8
Dielectric anisotropy	$\Delta\varepsilon=\varepsilon_{\parallel}-\varepsilon_{\perp}$	-4.2
Splay elastic constant	K_1	16.7 pN
Twist elastic constant	K_2	7.3 pN
Bend elastic constant	K_3	18.1 pN

MLC–6608 is a nematic LC with negative dielectric anisotropy. According to the supplier-provided material parameters, $\varepsilon_{\parallel}=3.6$, $\varepsilon_{\perp}=7.8$, giving a negative dielectric anisotropy of $\Delta\varepsilon=\varepsilon_{\parallel}-\varepsilon_{\perp}=-4.2$. Therefore, under an applied in-plane electric field, the director tends to reorient perpendicular to the electric-field direction. This field-induced reorientation competes with the homeotropic anchoring imposed by the VAPI boundaries and the molecule–air interface, contributing to the director distortion described in the main text. The elastic constants used in the director-field simulation are (K_1 = 16.7) pN, (K_2 = 7.3) pN, and (K_3 = 18.1) pN, corresponding to splay, twist, and bend deformations, respectively. The comparatively small (K_2) value suggests that twist deformation is more accessible than splay or bend deformation, which may facilitate the twist component of the observed pinwheel-like splay–bend–twist configuration.

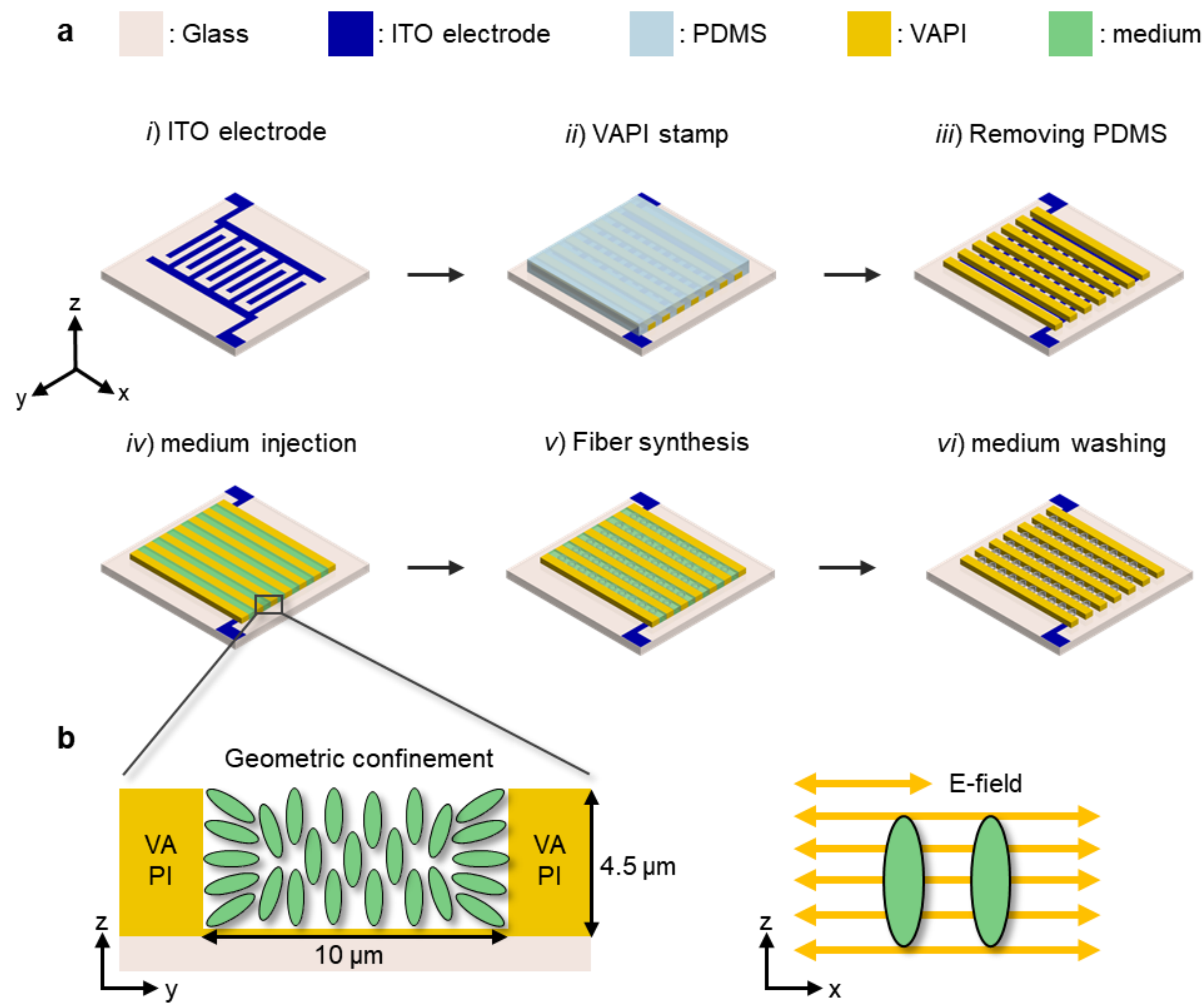

Supplementary Fig. 1. **Step-by-step fabrication process. a**, i) An indium tin oxide (ITO)-coated quartz substrate was sequentially cleaned with acetone, ethanol, and deionized water. ii) A pre-cleaned ITO substrate was coated with a vertically anchored polyimide (VAPI) using a patterned polydimethylsiloxane (PDMS) stamp to define the desired alignment region. iii) After the coating, the PDMS stamp was carefully peeled off, leaving a thin VAPI layer on the ITO surface. iv) A mesophase medium (MLC-6608, Merck) was then introduced into the patterned channel by capillary action at room temperature. v) Nanofibers were fabricated through the controlled chemical vapor deposition polymerization (CVP) process under a defined alternating-current (AC) electric field. The detailed experimental setup and conditions for CVP are provided in Supplementary Fig. 3. vi) After polymerization, the residual medium was completely removed by repeated washing with ethanol and hexane, yielding the desired chiral pinwheel-structured nanofibers on the ITO substrate. **b**, Schematic representation of the molecular orientation. The diagram shows the orientation behavior of the anisotropic molecules on the VAPI wall and at the air interface. The strong vertical anchoring at the VAPI surface and the air boundary induce a molecular orientation across the thin film (≈ 4.5 μm at the VAPI side and ≈ 10 μm at the air side). Under the applied AC electric field, the molecular dipoles align

predominantly along the z-direction, leading to anisotropic polarization that drives the helical or “pinwheel” organization within the nanofibers.

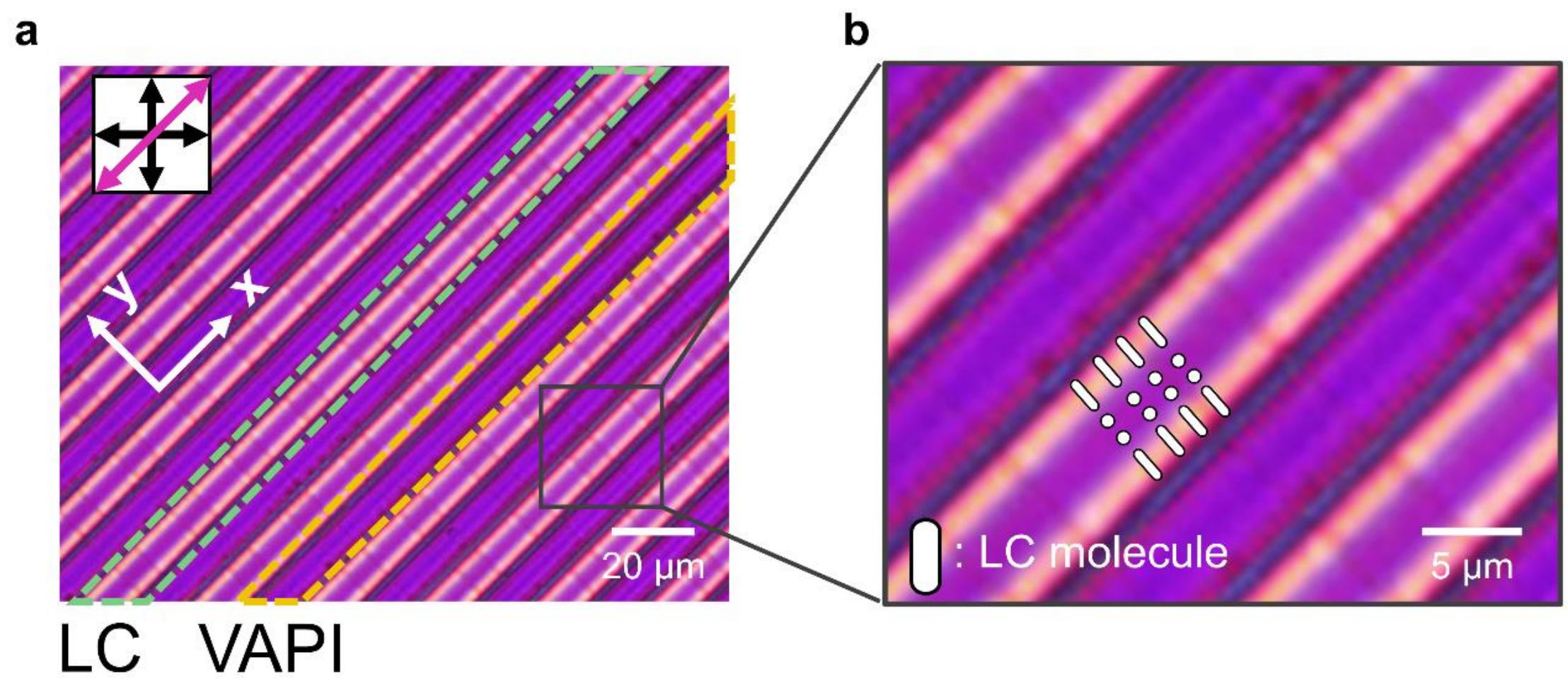

Supplementary Fig. 2. Polarized optical microscopy (POM) images of the mesophase under crossed polarizers with a quarter-wave plate. a, Low-magnification image showing the periodic alignment pattern of the nematic mesophase, liquid crystalline (LC) molecule, confined within vertically aligned polyimide (VAPI) channels. The director is oriented homeotropically in the LC region and normal to the VAPI sidewalls, forming a well-defined alignment contrast across the patterned substrate. **b**, High-magnification image revealing distinct birefringent colors: the central LC region appears magenta, corresponding to the homeotropic alignment along the z-axis, while the areas near the VAPI boundaries appear yellow due to vertical anchoring normal to the sidewalls. Scale bars: 10 µm.

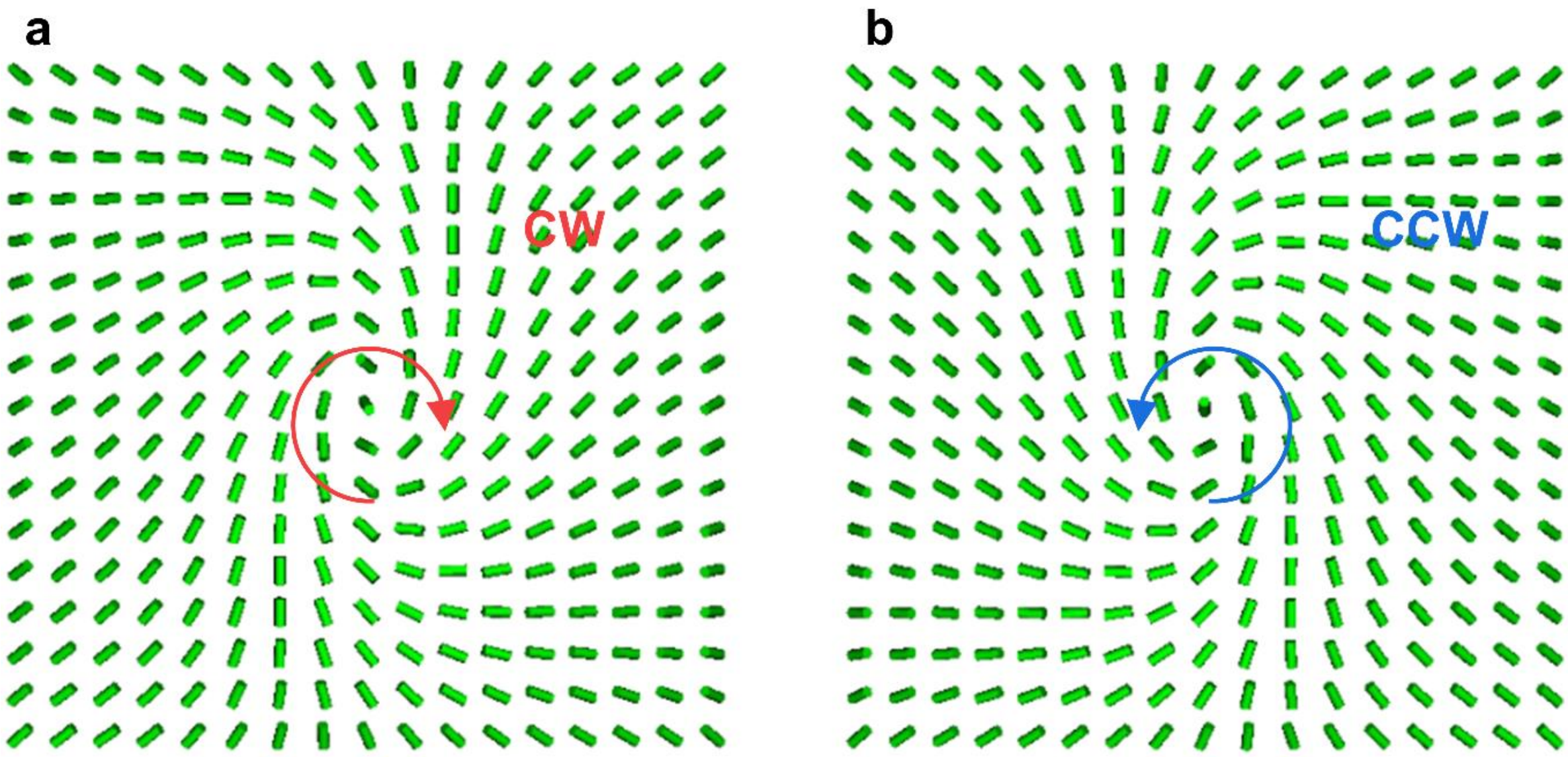

Supplementary Fig. 3. Numerical simulation of director-field configurations for pinwheel domains. Simulated in-plane director-field maps showing representative (**a**) clockwise (CW) and (**b**) counterclockwise (CCW) pinwheel configurations generated under the confined geometry and applied in-plane electric field. Green rods indicate the local orientation of the liquid crystal director. The simulated director field relaxes into a twist–spiral arrangement rather than a simple two-dimensional −1 point defect, supporting that the experimentally observed pinwheel texture originates from a three-dimensional splay–bend–twist distortion of the confined MLC–6608 mesophase. Red and blue arrows indicate the rotational sense of the CW and CCW domains, respectively.

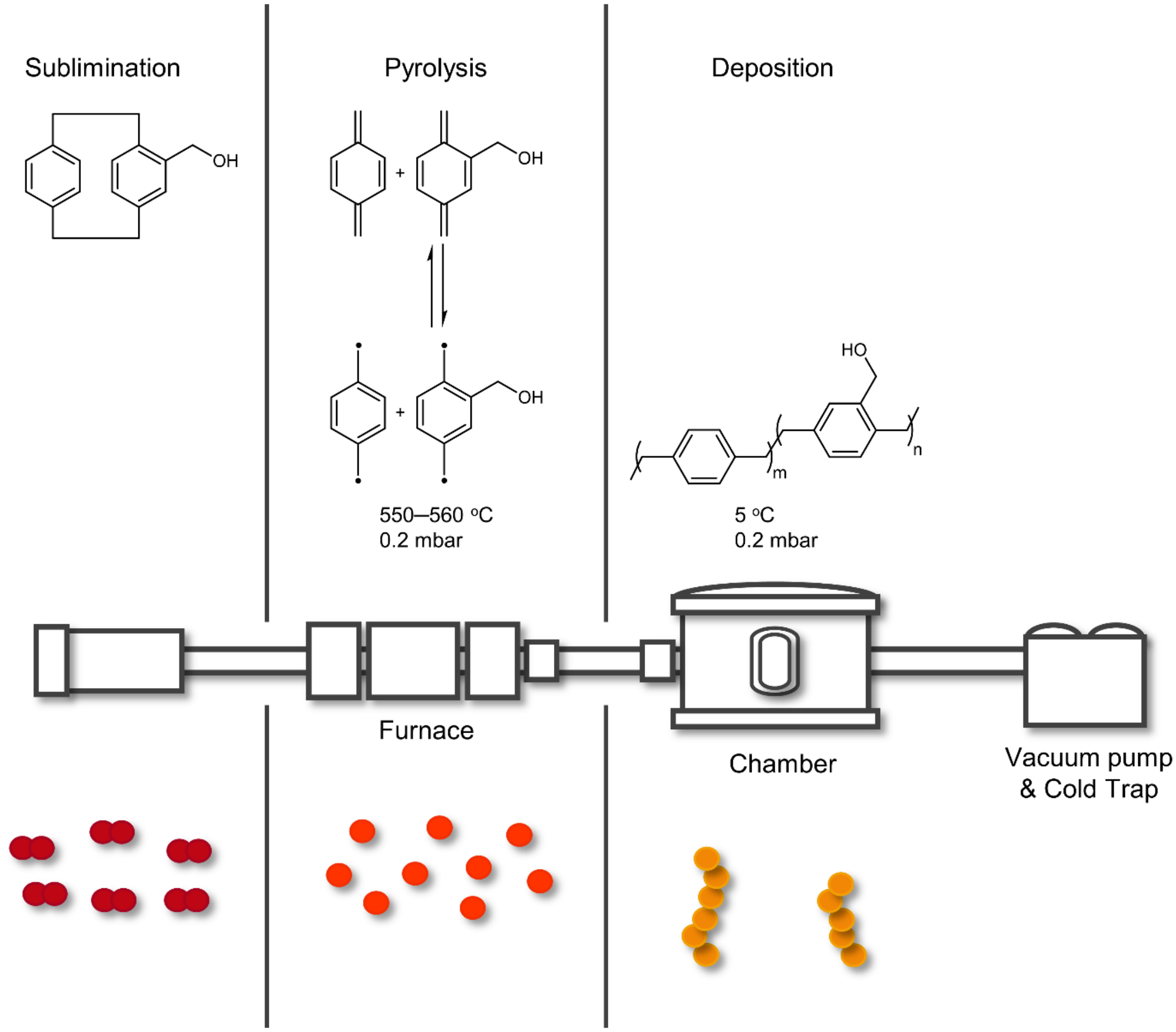

Supplementary Fig. 4. Schematic illustration of the CVP process used to form poly(hydroxymethyl-p-xylylene) (PPX–OH) nanofibers. The achiral monomer, 4-hydroxymethyl[2,2]paracyclophane (PCP–OH), undergoes a three-step process consisting of sublimation, pyrolysis, and deposition under reduced pressure (0.2 mbar). During sublimation, solid PCP–OH is vaporized and transferred into the furnace zone. In the pyrolysis region (550–560 °C), the monomer is thermally cleaved into reactive p-quinodimethane diradicals. These transient species are transported into the deposition chamber maintained at 5 °C, where they recombine and polymerize on the substrate surface to form PPX–OH thin films or nanofibers that replicate the underlying template structure. The system consists of a continuous vacuum line connected through a furnace and a cold trap, ensuring efficient transport of diradical intermediates and condensation exclusively at the cooled substrate region. Colored spheres schematically represent the transformation of the molecular species from dimeric PCP–OH (red) → reactive diradicals (orange) → polymerized PPX–OH chains (yellow).

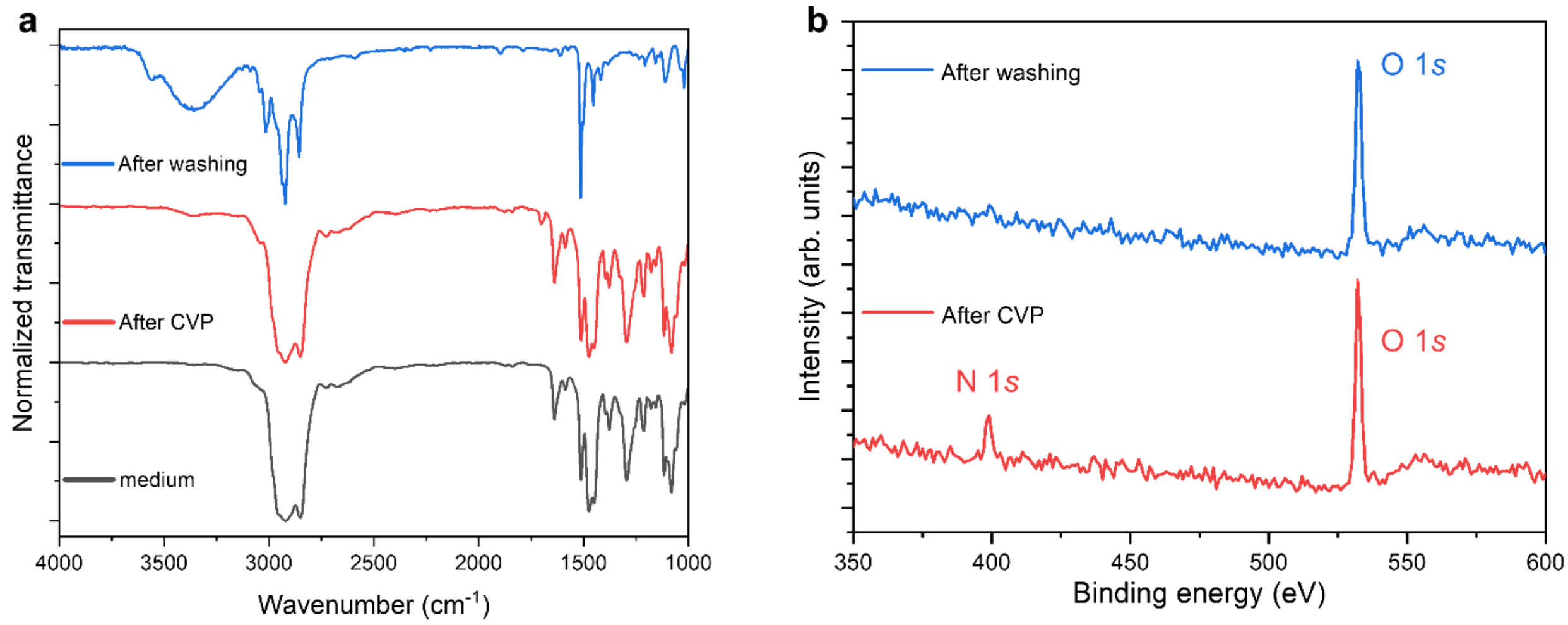

Supplementary Fig. 5. Structural confirmation of poly(hydroxymethyl-p-xylylene) (PPX–OH) formation and medium removal. a, Fourier transform infrared (FT-IR) spectra of the medium (black), after chemical vapor deposition polymerization (CVP) (red), and after washing (blue). The appearance of new absorption bands at 3300 cm^{-1} (O–H stretching) after CVP confirms the formation of PPX–OH. **b**, X-ray photoelectron spectroscopy (XPS) spectra after CVP (red) and after washing (blue). The N 1*s* peak (~399 eV) present after CVP vanishes completely after washing, while the O 1*s* signal (~532 eV) remains, further confirming that the medium and dopant were fully removed and only the polymer framework remains. Because the detailed chemical formula of MLC6608 (Merck) is not disclosed, the exact XPS peak assignments cannot be fully verified. However, the presence of an N 1*s* peak—without any cyano (C≡N) stretching signal in the FT-IR spectrum—suggests that the medium contains nitrogen but not a cyano functional group.

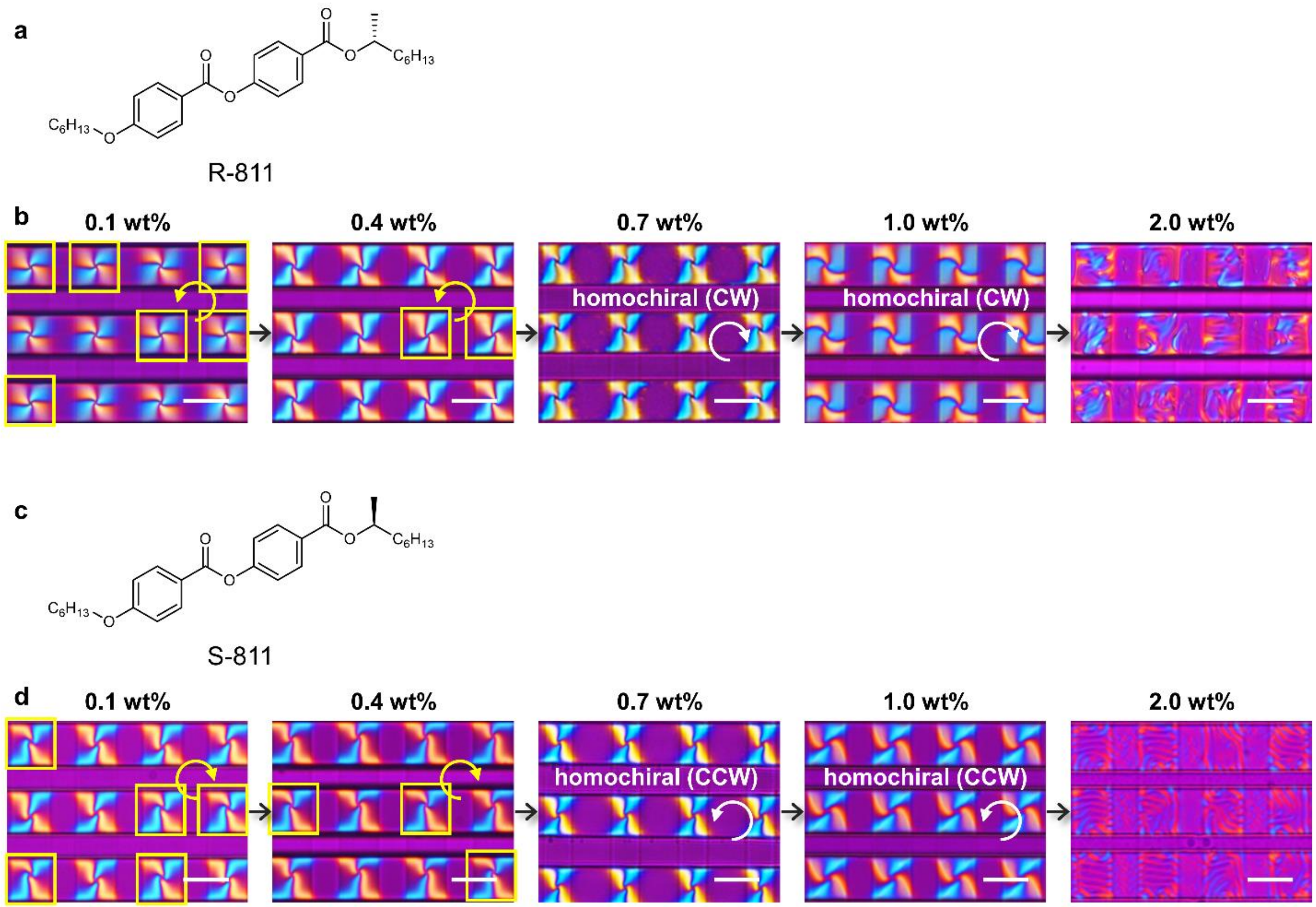

Supplementary Fig. 6. Concentration-dependent chiral control of field-induced pinwheel arrays. **a,c**, Molecular structures of R-811 and S-811 chiral dopants. **b,d**, Polarized optical microscopy (POM) images of MLC6608 doped with different concentrations of R-811 (**b**) and S-811 (**d**) under an applied electric field of 4.5 V μm^{-1}. At 0.1 and 0.4 wt%, incomplete handedness selection is observed, with minor oppositely handed pinwheel domains highlighted by yellow boxes. At 0.7 and 1.0 wt%, the doped systems form uniform homochiral pinwheel arrays: clockwise (CW) for R-811 and counterclockwise (CCW) for S-811. At 2.0 wt%, fingerprint-like cholesteric textures appear, indicating that excessive dopant loading promotes intrinsic cholesteric self-assembly rather than field-induced pinwheel formation. These results identify the concentration window in which the dopant effectively biases the handedness of field-induced symmetry breaking. Scale bars, 10 μm.

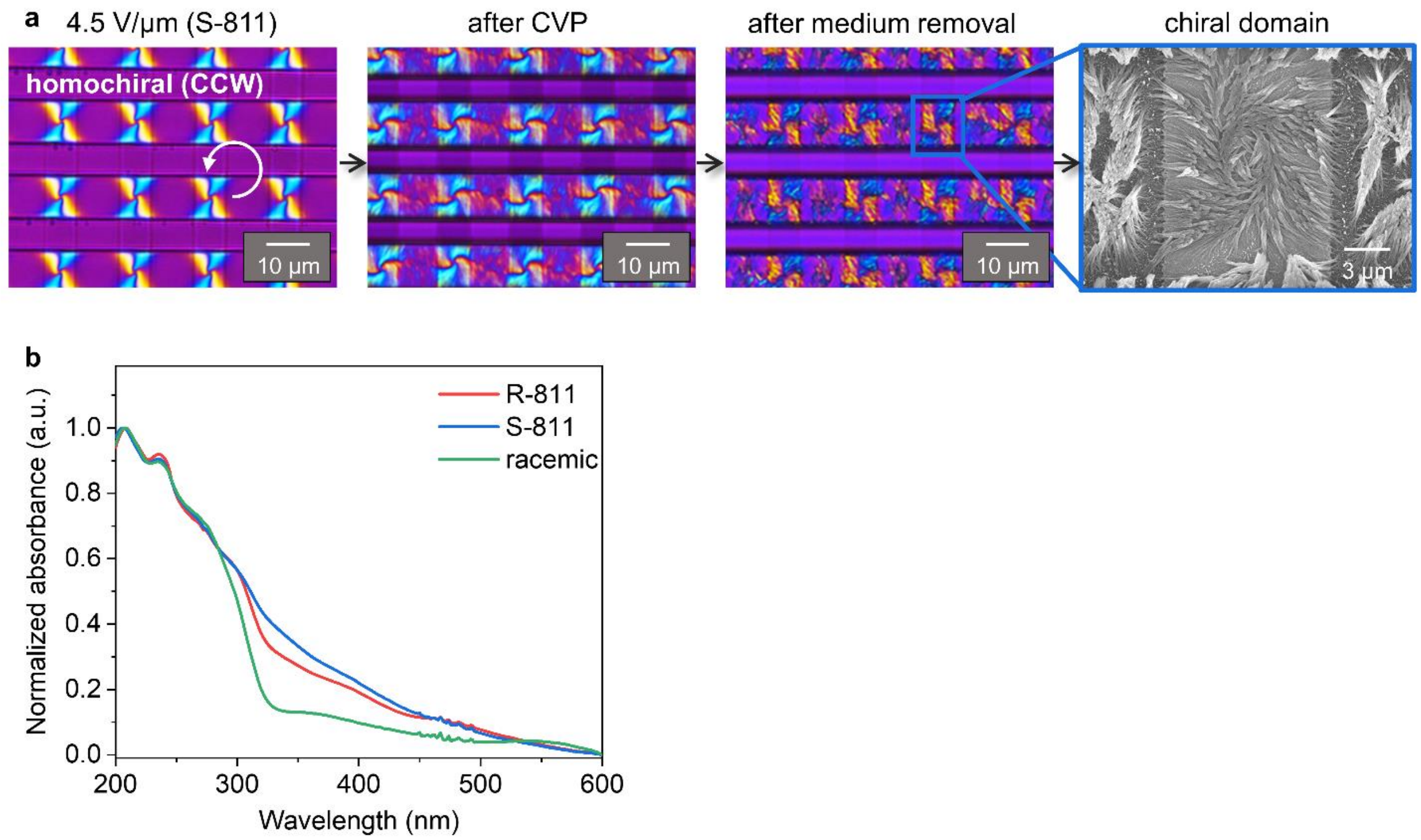

Supplementary Fig. 7. Homochiral formation of coumterclockwise (CCW) structures induced by S-811 dopant. **a**, Polarized optical microscopy (POM) images of the liquid crystal (LC) doped with S-811 under 4.5 V/μm, showing uniform CCW homochiral domains. The characteristic chiral texture is preserved after chemical vapor deposition polymerization (CVP) and remains unchanged after LC removal, confirming the stability of the transferred morphology. The rightmost scanning electron microscopy (SEM) image shows the corresponding CCW chiral nanofiber domain after LC removal. Scale bars, 10 μm (POM) and 3 μm (SEM). **b**, The chiral nanofibers exhibit distinct absorption bands below 280 nm, which are commonly observed in 4-hydroxymethyl[2,2]paracyclophane (PCP-OH) nanofibers prepared via the CVP process. These features arise from cooperative effects among the assembled chromophores, as reported in previous studies[1,2].

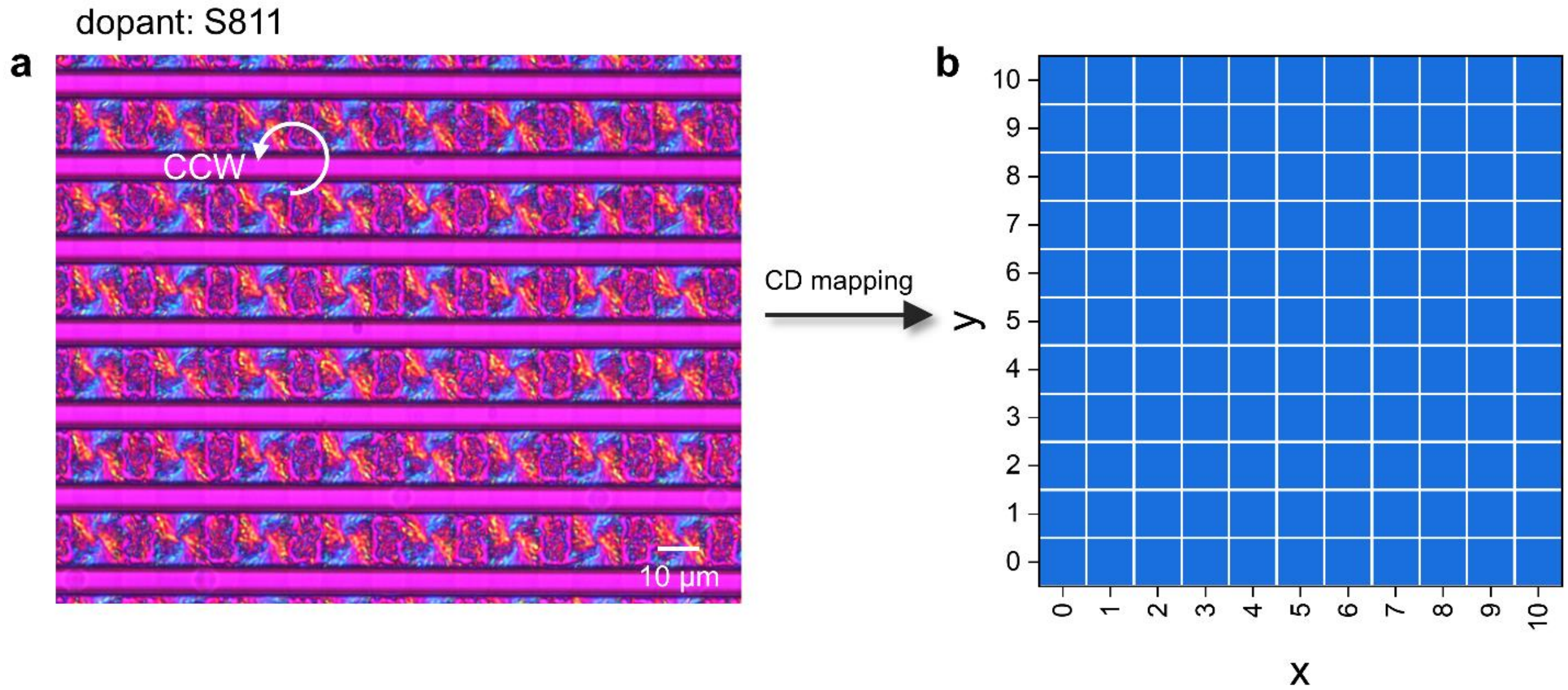

Supplementary Fig. 8. Focused circular dichroism (CD) mapping of S-811–induced homochiral domains. a, Polarized optical microscopy (POM) image of the S-811–doped medium after chemical vapor deposition polymerization (CVP), showing uniform CCW homochiral texture across the entire area. **b**, Corresponding spatial CD mapping acquired using a focused beam, where the entire region exhibits consistent negative ellipticity, confirming single-handed (counterclockwise (CCW)) chirality over the measured field. Scale bar, 10 μm.

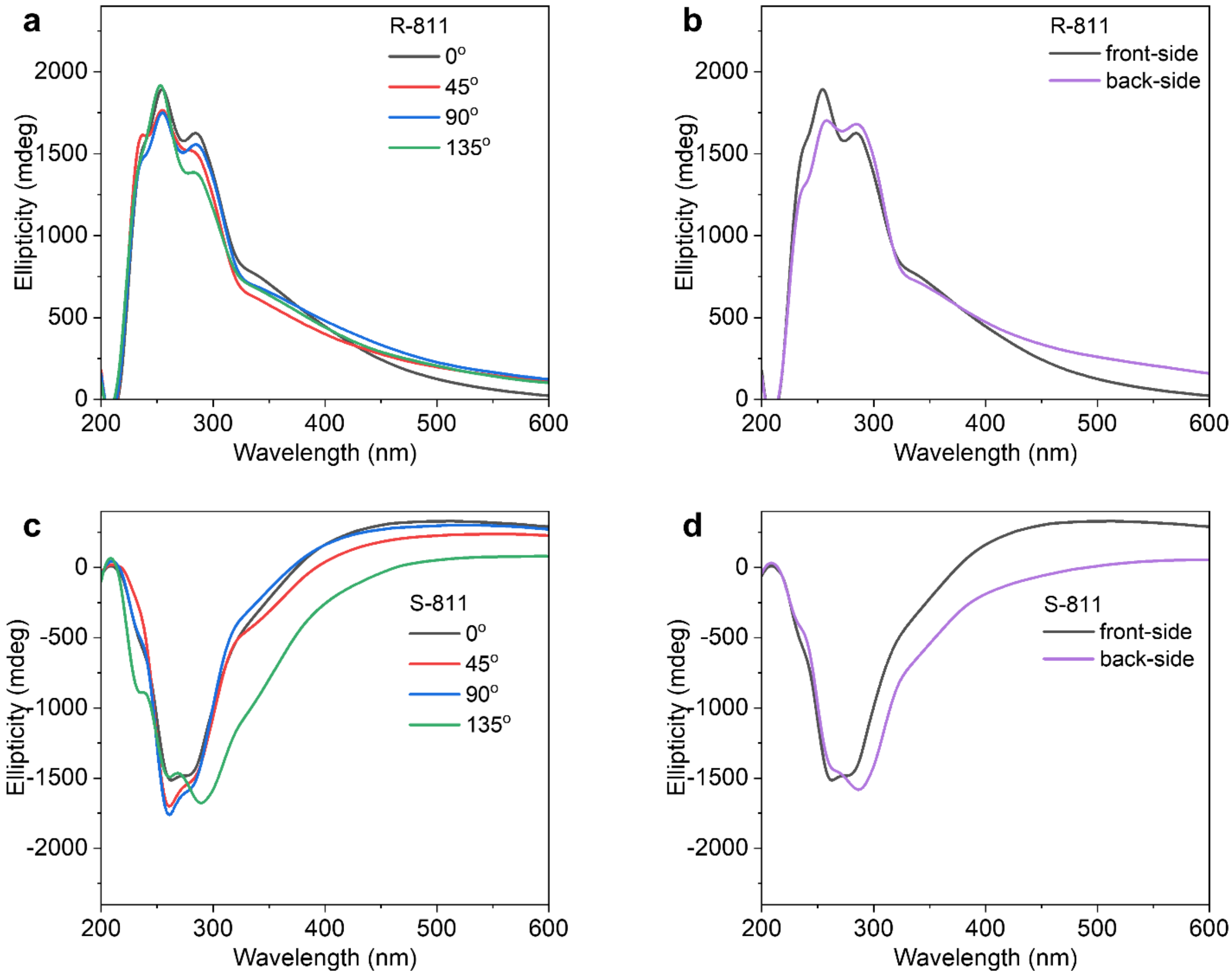

Supplementary Fig. 9. Orientation-dependent circular dichroism (CD) control measurements of R-811- and S-811-templated PPX–OH scaffolds. a,**c**, CD spectra of the R-811- and S-811-templated scaffolds measured after in-plane sample rotation at 0°, 45°, 90°, and 135°. The handedness-dependent CD sign and overall spectral shape are preserved at all rotation angles, indicating that the observed CD response is not dominated by sample-orientation-dependent linear dichroism or linear birefringence artifacts. **b**,**d**, Front- and back-side CD measurements of the R-811- and S-811-templated scaffolds. The spectra retain the same handedness-dependent sign and similar spectral features after flipping the sample, supporting that the CD response primarily originates from the chiral nanofiber architecture rather than from substrate- or measurement-geometry-induced artifacts.

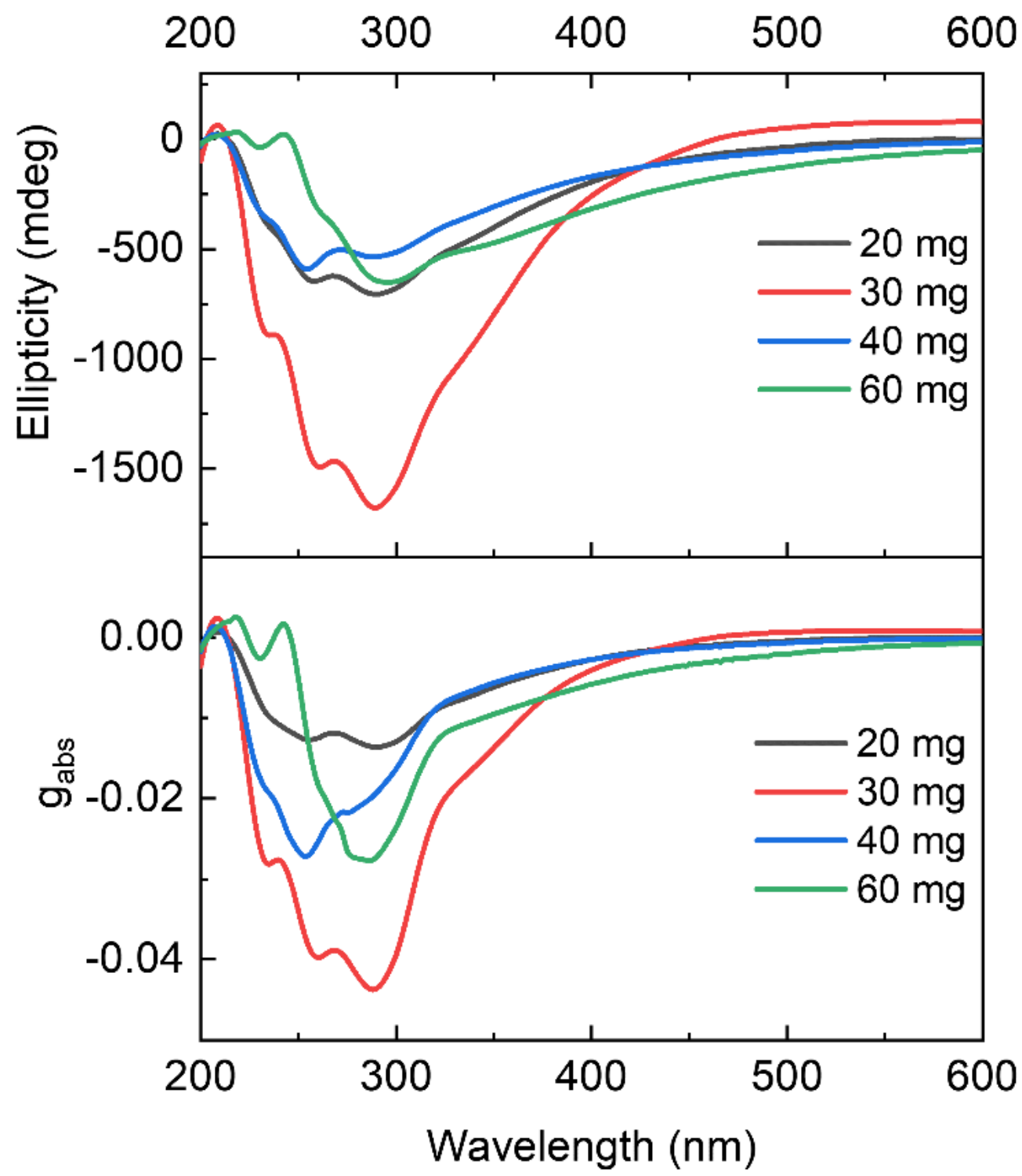

Supplementary Fig. 10. 4-hydroxymethyl[2,2]paracyclophane (PCP–OH) precursor amount-dependent circular dichroism (CD) response of S-811-templated poly(hydroxymethyl-p-xylylene) (PPX–OH) scaffolds. CD spectra and corresponding (g_{abs}) spectra of S-811-templated PPX–OH scaffolds prepared by chemical vapor deposition polymerization (CVP) using different PCP–OH precursor amounts. The CD intensity and (g_{abs}) values vary with precursor amount, with the 30 mg condition showing the strongest response. This indicates that the chiroptical magnitude is affected by the amount of deposited PPX–OH scaffold and/or nanofiber density. Despite this variation, all samples retain the same negative Cotton effect and similar spectral profiles, supporting that the handedness-dependent CD response is preserved across different scaffold thicknesses.

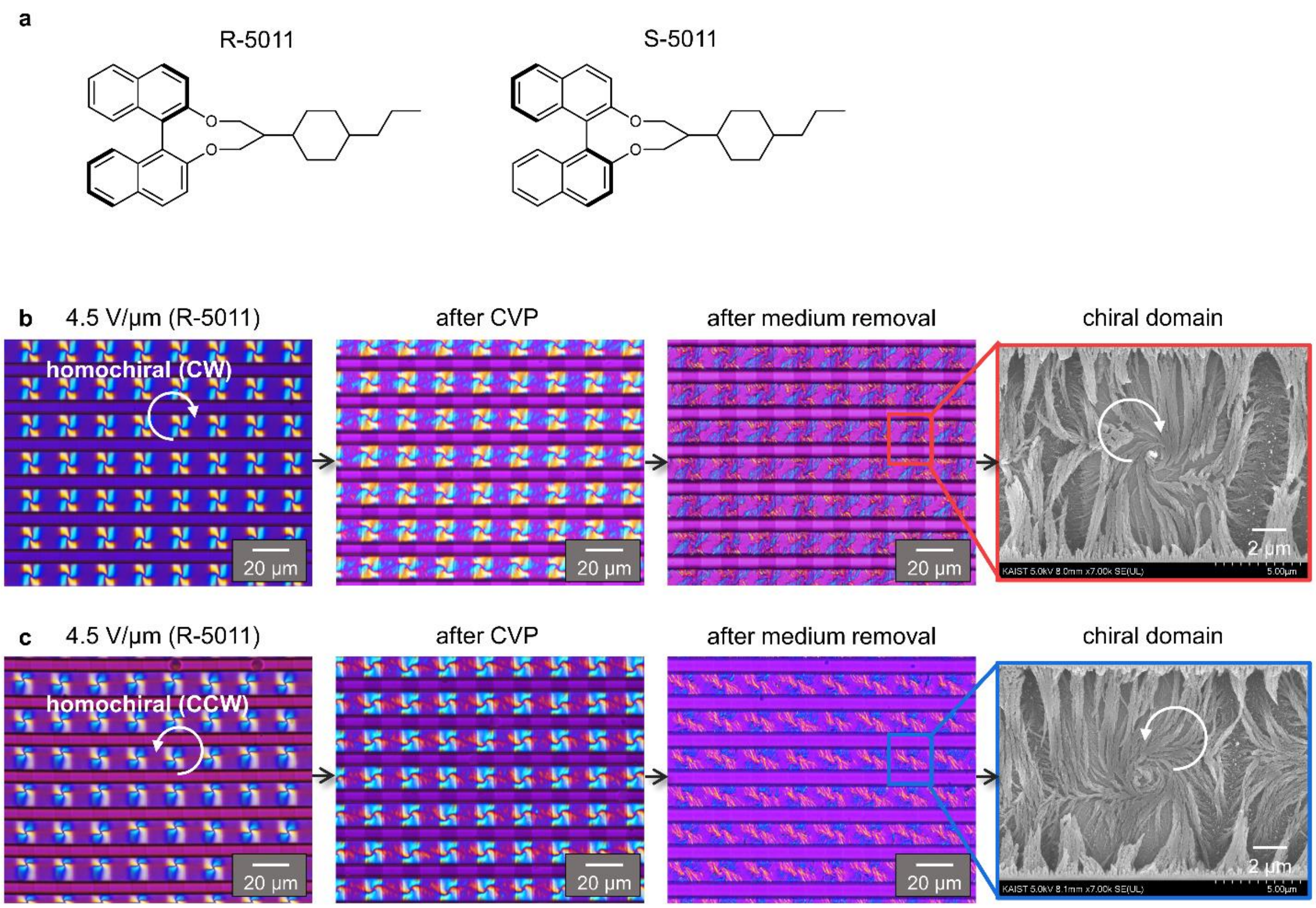

Supplementary Fig. 11. Homochiral domain formation using axially chiral dopant R/S-5011. a, Molecular structures of R-5011 and S-5011 chiral dopants possessing axial chirality. **b**, Polarized optical microscopy (POM) images of the R-5011–doped medium under 4.5 V/μm, showing uniform clockwise (CW) homochiral domains. The characteristic texture is retained after chemical vapor deposition polymerization (CVP) and remains unchanged after medium removal, indicating stable chirality transfer to the polymer scaffold. The corresponding scanning electron microscopy (SEM) image confirms the preserved CW helical nanofiber morphology. **c**, POM and SEM images of S-5011–doped medium showing counterclockwise (CCW) homochiral structures under identical conditions. The opposite handedness of the domains verifies that the induced chirality directly follows the enantiomeric form of the dopant. Scale bars, 20 μm (POM) and 5 μm (SEM).

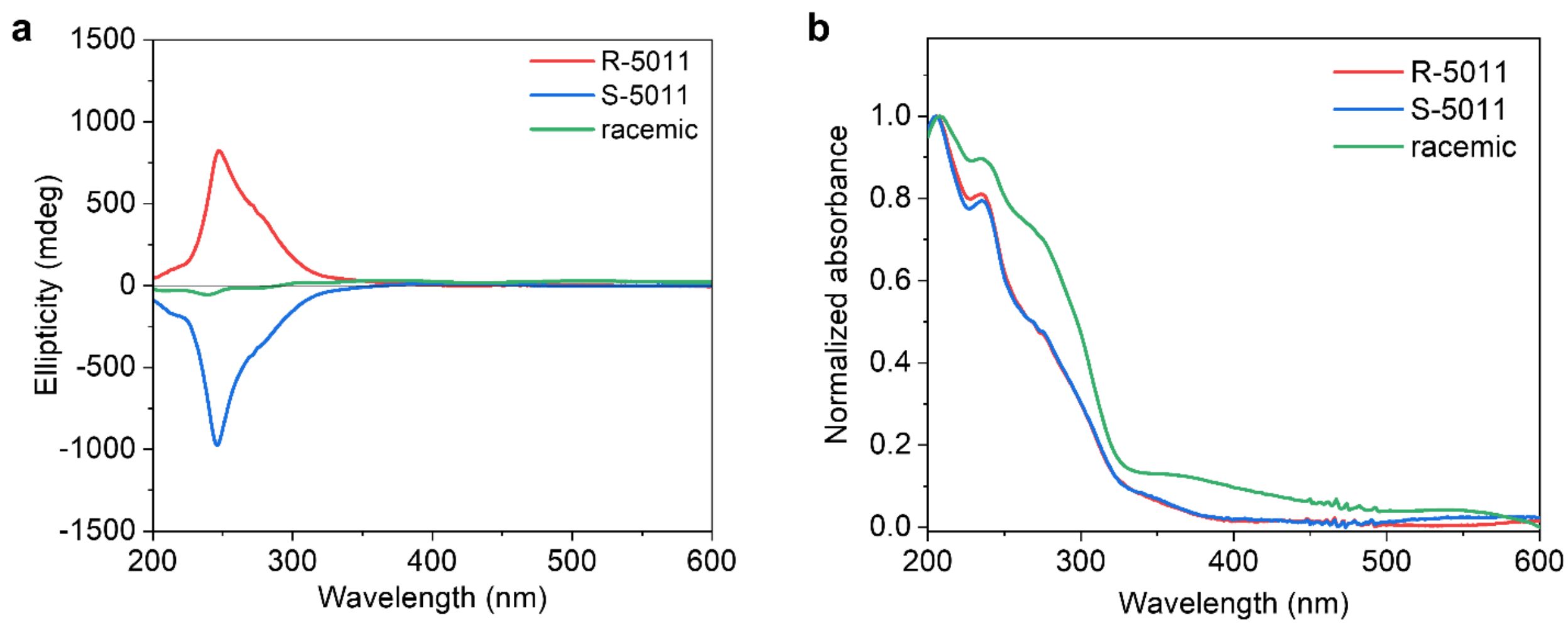

Supplementary Fig. 12. Circular dichroism (CD) and absorption spectra of R/S-5011–templated polymer nanofibers. a, CD spectra of polymer nanofiber films templated with R-5011 (red) and S-5011 (blue) after chemical vapor deposition polymerization (CVP) and medium removal, showing strong single-sign Cotton effects with opposite ellipticity, indicative of uniform clockwise (CW) and counterclockwise (CCW) homochirality, respectively. The undoped (racemic) film exhibits a flat baseline (green), confirming the absence of net optical activity. The mirror-image relationship between the R- and S-5011 spectra demonstrates that the macroscopic handedness directly reflects the molecular chirality of the dopant. **b**, Normalized absorption spectra of the polymer nanofiber films prepared with R-5011 (CW, red), S-5011 (CCW, blue), and without dopant (racemic, green). In fig. S8B, the chiral nanofibers exhibit distinct absorption bands below 280 nm, which are commonly observed in 4-hydroxymethyl[2,2]paracyclophane (PCP-OH) nanofibers prepared via the CVP process. These features arise from cooperative effects among the assembled chromophores, as reported in previous studies[1,2].

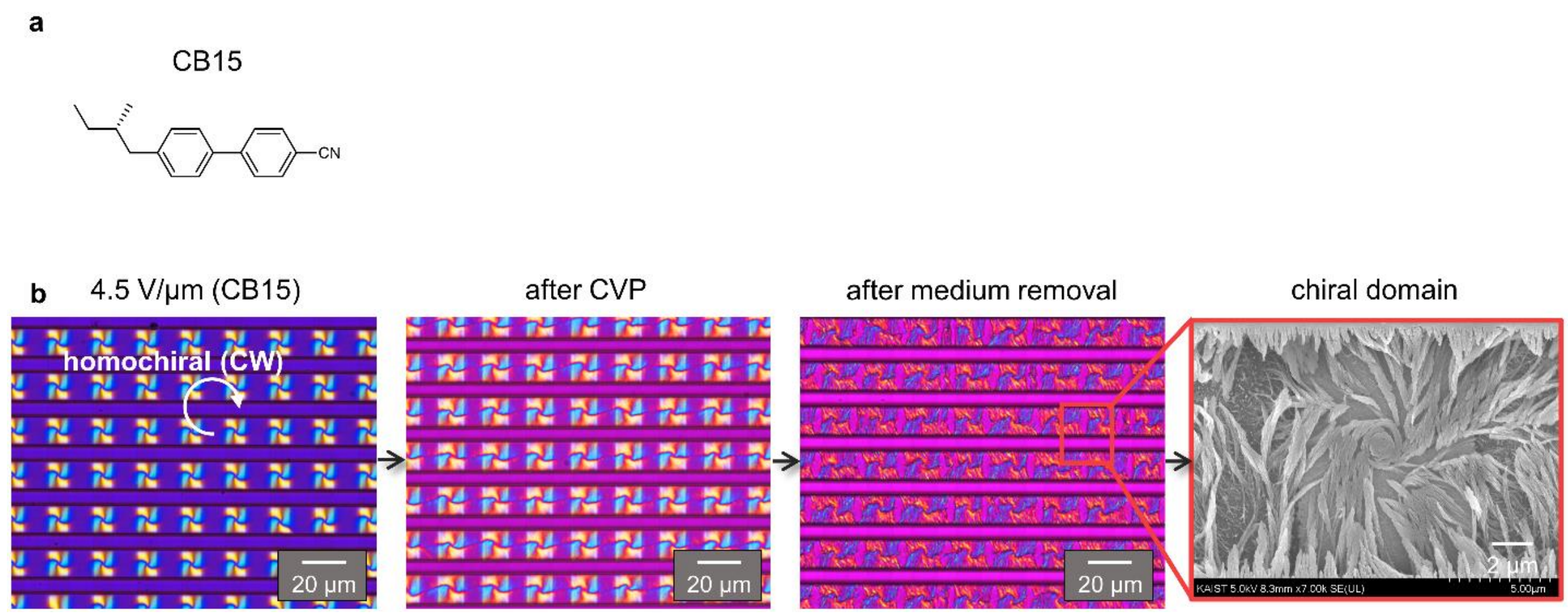

Supplementary Fig. 13. Homochiral domain formation using axially chiral dopant CB15. a, Molecular structure of CB15 exhibiting point chirality. **b**, Polarized optical microscopy (POM) images of the CB15-doped medium under 4.5 V/μm, showing the formation of uniform clockwise (CW) homochiral domains. The characteristic chiral texture is maintained after chemical vapor deposition polymerization (CVP) and remains unchanged after medium removal, confirming stable chirality transfer to the polymer framework. The corresponding scanning electron microscopy (SEM) image further verifies the preserved CW helical nanofiber morphology. Scale bars, 20 μm (POM) and 5 μm (SEM).

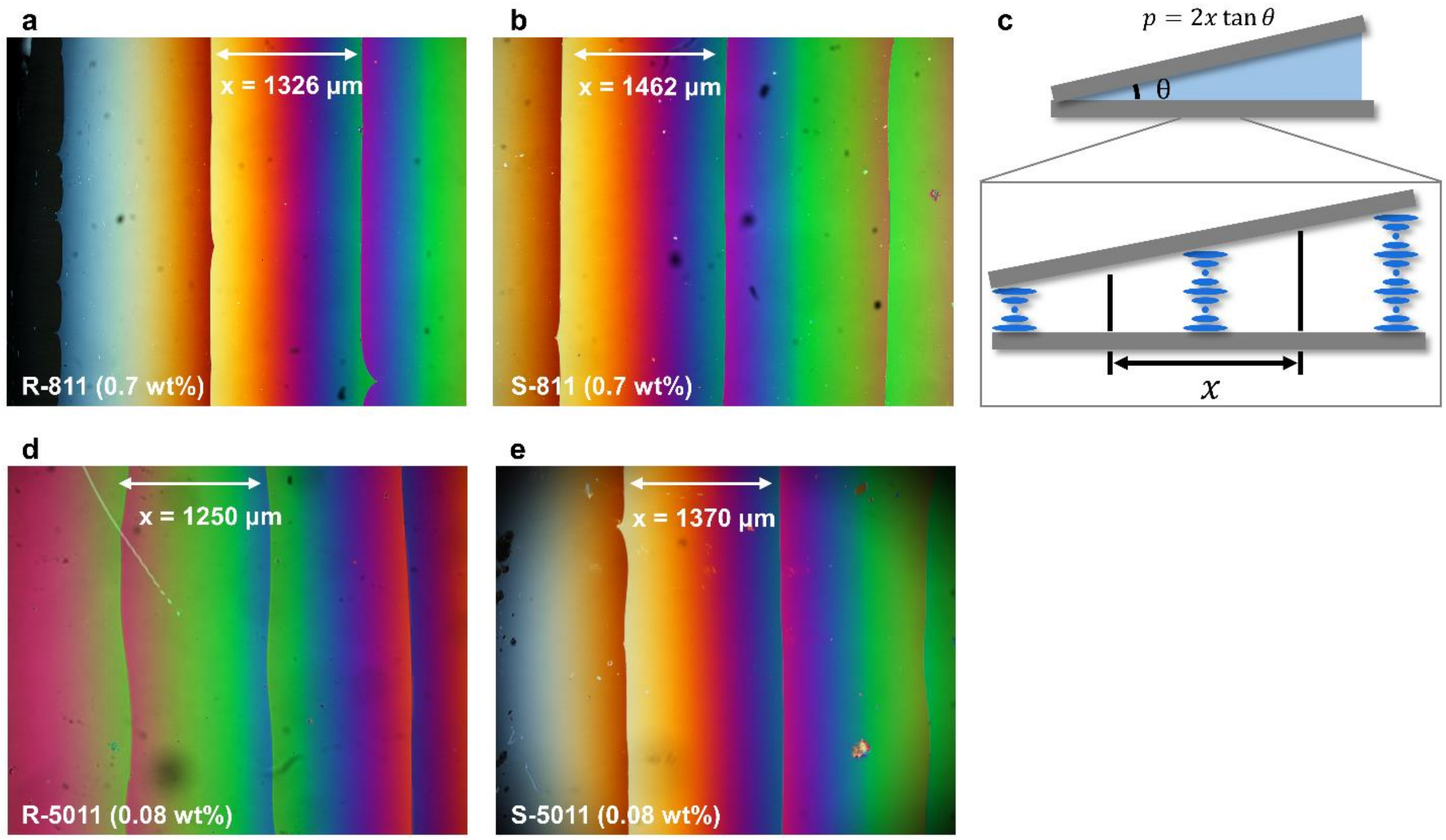

Supplementary Fig. 14. Cholesteric pitch measurements of R/S-811- and R/S-5011-doped MLC6608. **a,b,** Polarized optical microscopy images of wedge cells prepared with MLC6608 doped with 0.7 wt% R-811 (**a**) and 0.7 wt% S-811 (**b**). The distance between adjacent Grandjean lines, x, was measured to be 1326 and 1462 µm, respectively. **c,** Schematic illustration of the wedge-cell method used to determine the cholesteric pitch, where the pitch was calculated using $p = 2x\tan\theta$. **d,e,** Polarized optical microscopy images of wedge cells prepared with MLC6608 doped with 0.08 wt% R-5011 (**d**) and 0.08 wt% S-5011 (**e**). The measured x values were 1250 and 1370 µm, respectively. Despite the much lower dopant concentration, R/S-5011 produced a cholesteric pitch comparable to that of R/S-811 at 0.7 wt%, indicating that R/S-5011 provides a stronger effective chiral bias in the MLC6608 mesophase.

Planar-rubbed alignment layers were used to impose a uniform helical structure in the cholesteric liquid crystal (CLC), with the helical axis normal to the substrates. A wedge cell was formed by slightly tilting the top substrate, so that the cell thickness increases linearly along the wedge direction. CLC must accommodate an integer number of half-turns across the cell gap; spatial variations produce discrete changes in the helical winding number. The corresponding phase boundaries appear in polarized optical microscopy (POM) as Grandjean lines running perpendicular to the rubbing direction. The thickness increment between adjacent lines is equal to half the pitch. Therefore, the local thickness increment is defined as $p = 2x\tan\theta$. The wedge angle θ was determined during cell fabrication using a 100 µm spacer tape. Specifically, $\tan\theta$ was obtained from the ratio of the spacer thickness (100 µm) to the in-plane distance between the tape edge and the line of substrate contact. With the measured $\tan\theta$ and the POM-derived Grandjean line spacing x, the cholesteric pitch p was calculated from the equation above.

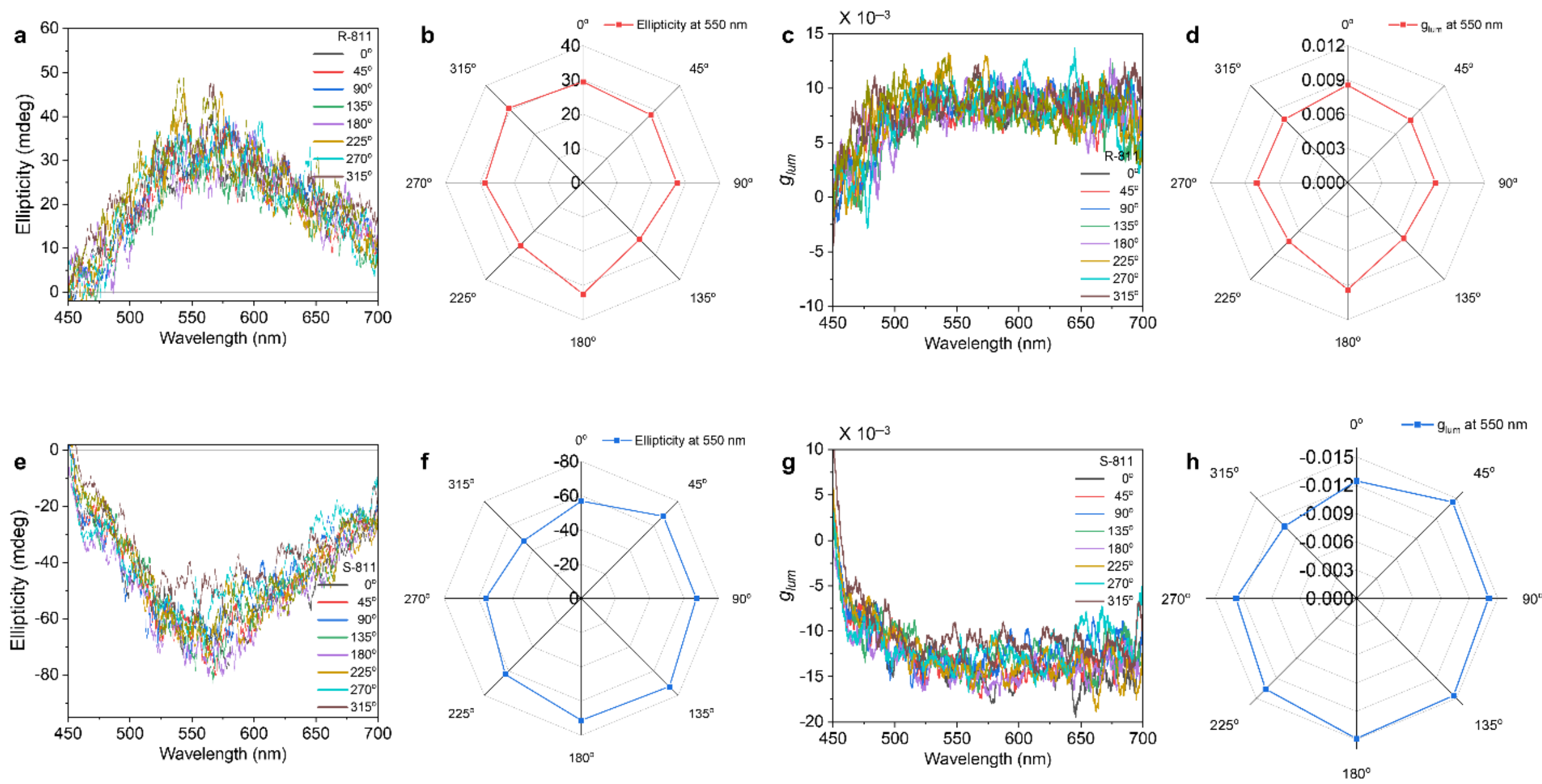

Supplementary Fig. 15. Angle-dependent circularly polarized luminescence (CPL) control measurements of dye-coated R-811- and S-811-templated poly(hydroxymethyl-p-xylylene) (PPX–OH) scaffolds. a,e, CPL spectra of dye-coated PPX–OH scaffolds templated by (**a**) R-811 and (**e**) S-811, measured during in-plane sample rotation from 0° to 315° in 45° steps. The R-811-templated scaffold retains positive CPL at all rotation angles, whereas the S-811-templated scaffold retains negative CPL. **b,f,** Polar plots of CPL ellipticity at 550 nm as a function of sample-rotation angle. **c,g,** Corresponding g_{lum} spectra measured at each rotation angle. **d,h,** Polar plots of g_{lum} at 550 nm. Although the ellipticity and g_{lum} values show moderate angular variation, the CPL sign and overall spectral shape are preserved for each handed scaffold, supporting that the observed CPL response is not dominated by sample-orientation artifacts.

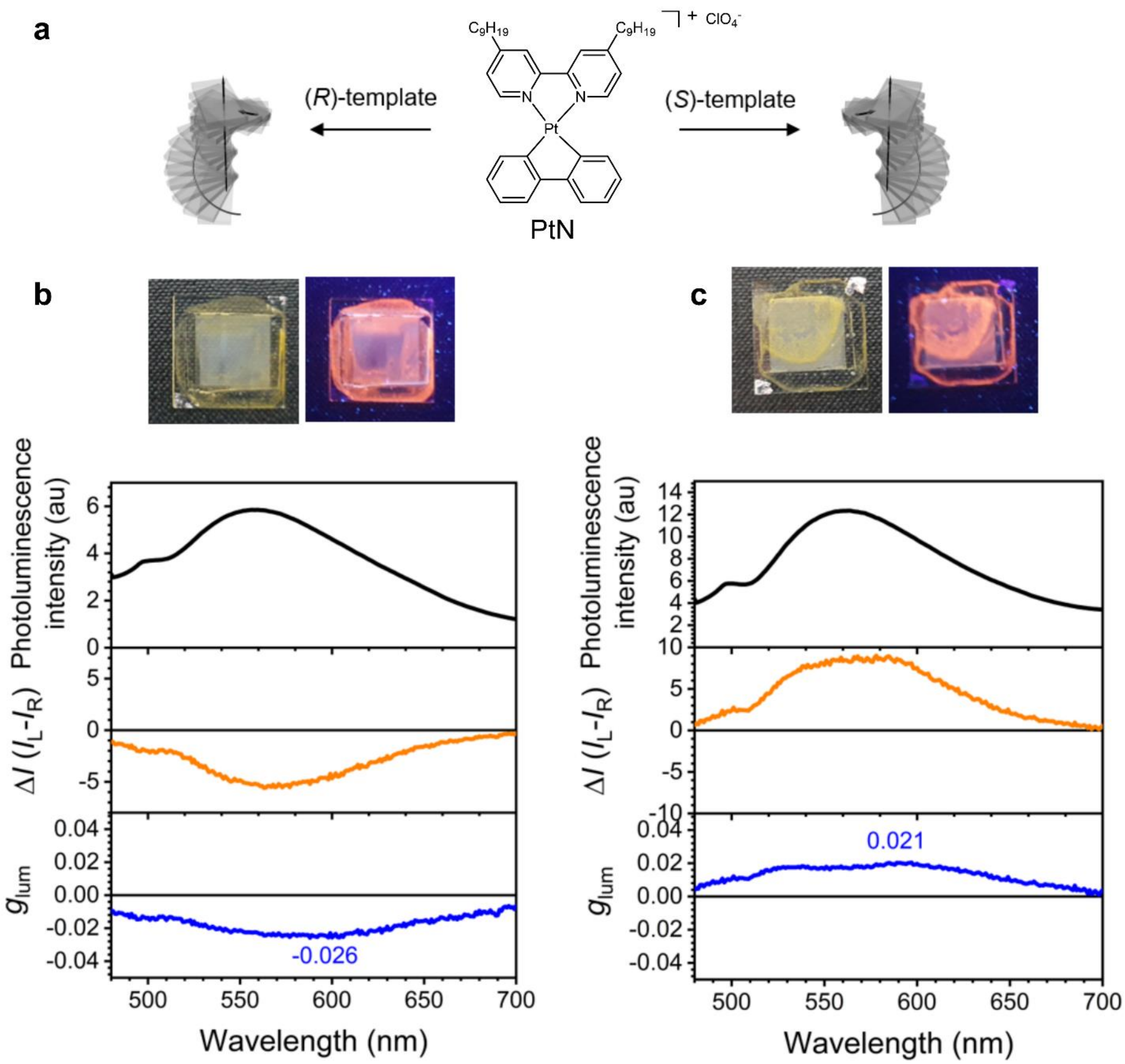

Supplementary Fig. 16. Circularly polarized luminescence (CPL) of PtN self-assemblies. a, Schematics for the formation of helical self-assemblies of PtN. **b**,**c**, Top, photos showing PtN self-assemblies produced on top of chiral templates visualized under room light (left) and 365 nm (right); bottom, photoluminescence (top), CPL (middle), and g_{lum} (bottom) spectra of PtN self-assemblies on (*R*)- (**b**) and (*S*)-templates (**c**). The values colored in blue are peak g_{lum}.

Synthetic Procedures

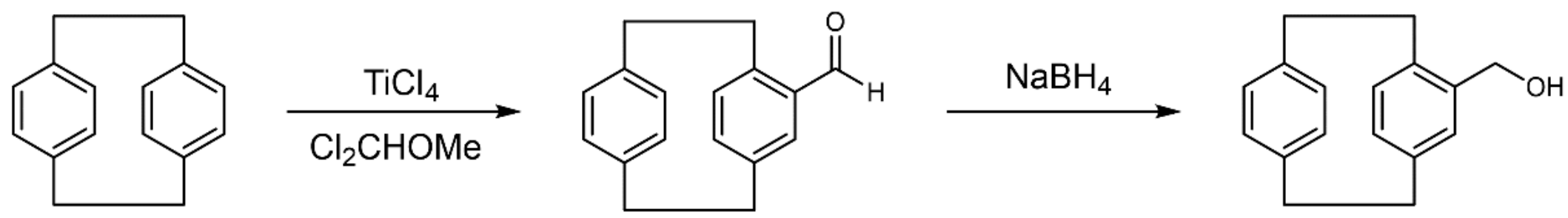

PCP PCP-CHO PCP-OH

Supplementary Fig. 17. Synthetic scheme for PCP-OH.

Synthesis of 4-formyl[2.2]paracyclophane (PCP-CHO): [2.2]Paracyclophane (PCP) (5 g, 24.0 mmol) was dissolved in dichloromethane (200 mL) and cooled to 0 °C. Titanium(IV) chloride (4.6 mL, 41.5 mmol) was added dropwise, and the reaction mixture was stirred for 1 h. 1,1-Dichlorodimethyl ether (3.6 mL, 39.3 mmol) was then added dropwise, and the mixture was stirred for an additional 4 h. The reaction was quenched with water, and the organic layer was separated, extracted with dichloromethane, and washed with saturated NaCl solution. The combined organic layers were dried over Na_2SO_4, filtered, and concentrated under reduced pressure. The crude product was purified by silica gel chromatography (n-hexane/ethyl acetate) to afford 4-formyl[2.2]paracyclophane (PCP-CHO) as white crystals (4.8 g, 89%).

- ^{1}H NMR (600 MHz, $CDCl_3$, δ): 9.87 (s, 1H), 6.94 (d, J = 2.0 Hz, 1H), 6.65 (dd, J = 7.8, 2.0 Hz, 1H), 6.52 (d, J = 7.8 Hz, 1H), 6.49 (dd, J = 7.9, 1.9 Hz), 6.42 (dd, J = 7.8, 1.9 Hz,), 6.36 (dd, J = 7.8, 1.9 Hz, 1H), 6.30 (dd, J = 7.8, 1.9 Hz, 1H), 4.05–4.01 (m, 1H), 3.21–2.85 (m, 7H).
- ^{13}C NMR (151 MHz, $CDCl_3$, δ): 191.94, 143.24, 140.67, 139.52, 139.46, 138.09, 136.61, 136.35, 136.13, 133.27, 132.93, 132.38, 132.17, 35.28, 35.16, 35.01, 33.63.

Synthesis of 4-formyl[2.2]paracyclophane (PCP-OH): PCP-CHO (3.7 g, 15.7 mmol) was dissolved in methanol (100 mL) and THF (10 mL). Sodium borohydride (2.4 g, 37.0 mmol) was added carefully, and the reaction mixture was stirred at room temperature for 6 h. The solvents were removed under reduced pressure, and the residue was taken up in dichloromethane (100 mL), washed with saturated NaCl solution, and dried over Na_2SO_4. After filtration and concentration, the crude product was purified by silica gel chromatography (n-hexane/ethyl acetate) to give 4-hydroxymethyl[2.2]paracyclophane (PCP-OH) as white crystals (3.7 g, 84%).

- ^{1}H NMR (600 MHz, $CDCl_3$, δ): 6.63 (dd, J = 7.8, 1.9 Hz, 1H), 6.57–6.49 (m, 4H), 6.42–6.41 (m, 2H), 4.81 (t, J = 5.6 Hz, 1H), 4.73 (d, J = 12.6 Hz, 1H), 4.41 (d, J = 12.6 Hz, 1H), 3.43 (ddd, J = 12.8, 10.0, 2.4 Hz, 1H), 3.19–3.01 (m, 6H), 2.92–2.87 (m, 1H).

^{13}C NMR (151 MHz, CDCl3, δ): 140.30, 139.73, 139.55, 139.26, 137.49, 135.02, 133.34, 133.24, 132.38, 132.17, 132.12, 129.05, 64.53, 35.29, 35.05, 34.40, 32.81.

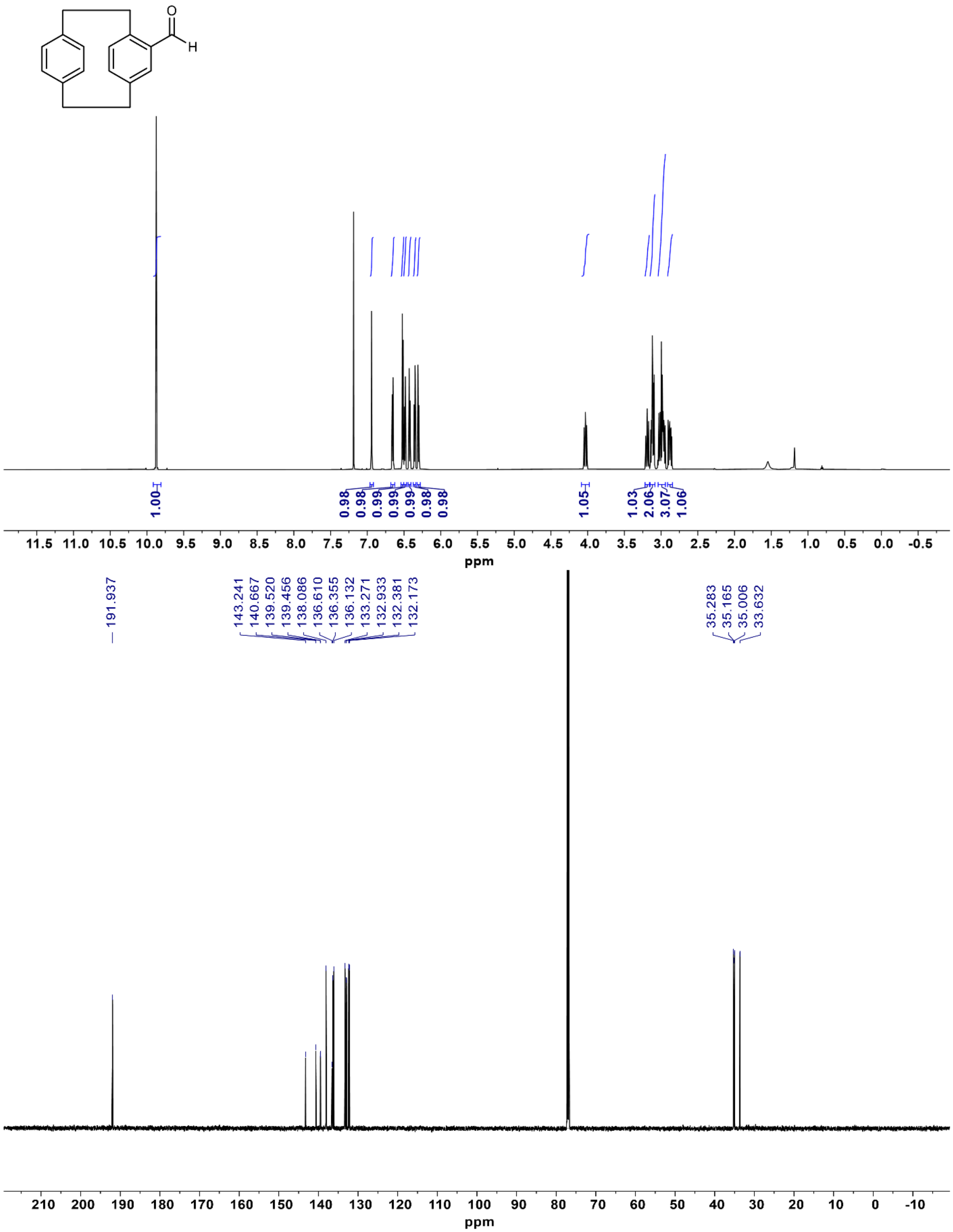

Supplementary Fig. 18. ^{1}H NMR spectrum of 4-formyl[2.2]paracyclophane (PCP-CHO) (600 MHz, top) and ^{13}C NMR spectrum (151 MHz, bottom) in $CDCl_3$.

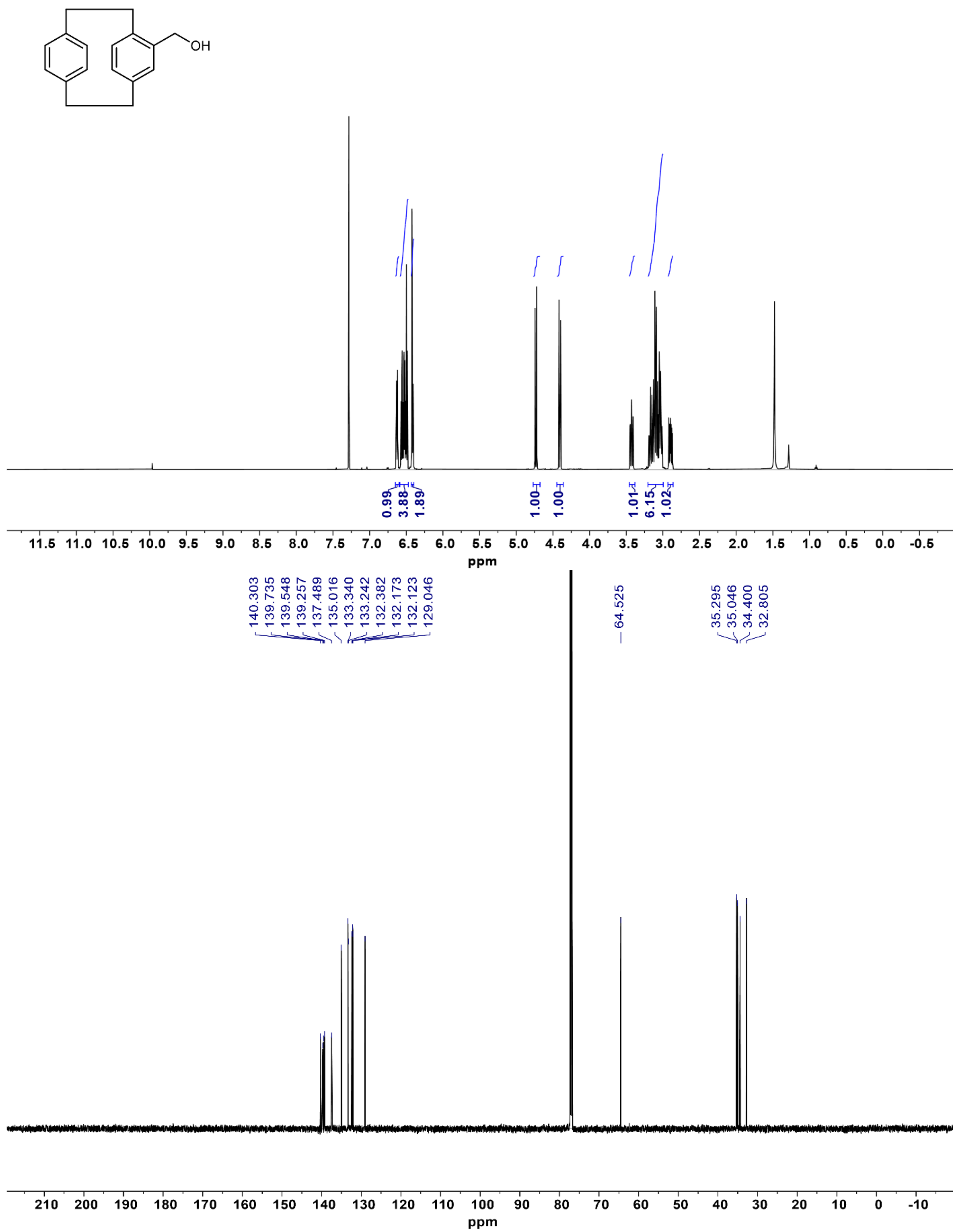

Supplementary Fig. 19. ^{1}H NMR spectrum of 4-hydroxymethyl[2,2]paracyclophane (PCP-OH) (600 MHz, top) and ^{13}C NMR spectrum (151 MHz, bottom) in $CDCl_3$.

References

1. Cheng, K. C. K. *et al.* Templated nanofiber synthesis via chemical vapor polymerization into liquid crystalline films. *Science (80-.).* **362**, 804–808 (2018).

2. Varadharajan, D. *et al.* Surfaces Decorated with Enantiomorphically Pure Polymer Nanohelices via Hierarchical Chirality Transfer across Multiple Length Scales. *Adv. Mater.* **34**, 2108386 (2022).
