# Supplementary material for: Microchiral pinwheel arrays based on achiral molecules
Source: Nat Commun. 2026 Aug 5;17:9413. doi: 10.1038/s41467-026-76089-z (PMC13538584; doi:10.1038/s41467-026-76089-z)
Supplement: Supplementary file 2 [file 41467_2026_76089_MOESM2_ESM.pdf]

nature portfolio

Peer Review File

Microchiral pinwheel arrays based on achiral molecules

Corresponding Author: Professor Dong Ki Yoon

This file contains all reviewer reports in order by version, followed by all author rebuttals in order by version.

Version 0:

Reviewer comments:

Reviewer #1

(Remarks to the Author)
Yoon et al. reported that a chiral mesophase, including a racemic one, can be modulated by an electric field, and that polymerization of cyclophane molecules within this mesophase enables transfer of the mesophase chirality to the resulting polymer. They further demonstrated that the use of chiral dopants allows the formation of optically active mesophases; in such cases, micrometer-scale macroscopic chirality is induced in the polymer, and this chirality can also be transferred to an achiral luminescent dye. Ultimately, circularly polarized luminescence is generated. The generality of this chirality-transfer strategy is examined using multiple chiral dopants and luminescent dyes. The resulting structures are characterized by POM, SEM, IR, XPS, CD, and related methods. Although the results are interesting and potentially important, several issues remain unresolved and should be addressed before the manuscript can be considered for acceptance.

1. The rationale for using a cyclophane-based polymer is unclear. Why did the authors choose a cyclophane-derived polymer in this study? Since this is not a common polymer system, the authors should clarify whether there is a specific reason why this material is essential, or whether it offers particular advantages over more conventional polymers. If the authors wish to claim broad applicability of their approach, transfer to a more general polymer system should at least be discussed, if not experimentally demonstrated.
2. The meaning of using different concentrations for different dopants is unclear. The manuscript employs different concentrations depending on the chiral dopant, but the reason for doing so is not adequately explained. A systematic study of the concentration dependence of the chiral dopant should be provided. In particular, at lower dopant concentrations, is it possible that a non-racemic mixture of right- and left-handed helices is formed? This point deserves discussion.
3. The relationship between macroscopic helicity and molecular-level chirality remains unclear. SEM images indicate the formation of micrometer-scale helical structures, but it is not clear whether molecular- or supramolecular-level chirality is also induced within the polymer. Is the polymer essentially amorphous, or does it possess any local ordered structure? This point should be discussed in greater depth.
4. The composition of the mesophase and thickness dependence should be clarified. The manuscript should more clearly describe what components constitute the mesophase. In addition, the dependence of CD and CPL intensity on the film thickness of both the mesophase and the polymer-containing complex should be investigated and discussed. Such analysis is necessary to distinguish intrinsic chiral optical responses from possible thickness-dependent optical artifacts.
5. Although CD and CPL are reported in the solid state, there is essentially no discussion of anisotropy-related artifacts. For solid-state CD measurements, the possible contributions of linear dichroism and linear birefringence should be carefully examined. For CPL measurements, possible contamination from CD as well as the effects of selective scattering and selective reflection should also be thoroughly evaluated. At present, the reliability of these optical data is not fully guaranteed.
6. For samples that are expected to be enantiomeric counterparts, the CD spectra should normally exhibit clear mirror-image relationships. The fact that the spectra in Fig. 2h do not do so suggests that factors such as differences in film thickness, sample orientation, scattering, or incomplete enantiomeric inversion may be involved. The authors should explain this point.
7. The chemical structures of the dopants should be shown in the manuscript.

Overall, this study presents an intriguing strategy for chirality transfer through mesophases and for the generation of circularly polarized luminescence. However, the points raised above should be adequately addressed through further explanation and, where necessary, additional experiments before the manuscript can be considered suitable for publication.

Reviewer #2

(Remarks to the Author)
The manuscript reports preparation and characterization of chiral structure from achiral building blocks, based on electric-field generated array of defects in director field. Originally racemic array can be turned into homochiral state by using chiral dopant, which breaks degeneracy of enantiomeric structures. Authors used a nematic LC confined in regular system of microchannels prepared from VAPI, which was exposed to in-plane electric field generated by comb-like electrodes. Under such condition, on the electrodes the pinwheel defects were generated, which can be used as templates to generate stable chiral arrangement of polymer fibers by CVP. Authors convincingly demonstrated possibility of obtaining chiral structures, e.g for generating of circularly polarized luminescence from achiral dye.
In general, the subject is interesting, and reported results might deserve publication in NatComm. However, few points should be clarified/presented in more clear way:
-mechanism of chiral defect creation is not clearly described, why pinwheel arrangement is favored over simple point -1 defects? The equations provided in SM are very general and to not provide the mechanism.
-I would suggest to give more detailed scheme of 3D director field in the defect region
-Figure S1b suggest that the used LC has a negative dielectric anisotropy -is it true? The data regarding dielectric anisotropy and elastic constants of the material should be provided
-direction o coordination axis system presented in Figure S1 do not gree with description of walls and electrodes direction (see line 74,75)
-concentration of used chiral dopany (~2%) should not be refered to as ultralow, its rather standard for commercial mixtures.

Version 1:

Reviewer comments:

Reviewer #1

(Remarks to the Author)
The authors have carefully addressed the concerns raised in the previous review. The revised manuscript provides substantially improved clarification of the rationale for the polymer system, the concentration dependence of the chiral dopants, the relationship between mesoscale helicity and molecular chirality, and the origin of the observed chiroptical responses. The additional experiments, control studies, and analyses significantly strengthen the manuscript and support the authors' interpretations. I believe that that the manuscript is now suitable for publication in Nature Communications.

Reviewer #2

(Remarks to the Author)
I have found responses provided in rebuttal letter satisfactory, and thus recommend publication of the manuscript in its current form.

Open Access This Peer Review File is licensed under a Creative Commons Attribution 4.0 International License, which permits use, sharing, adaptation, distribution and reproduction in any medium or format, as long as you give appropriate credit to the original author(s) and the source, provide a link to the Creative Commons license, and indicate if changes were

made.
In cases where reviewers are anonymous, credit should be given to 'Anonymous Referee' and the source.
The images or other third party material in this Peer Review File are included in the article's Creative Commons license, unless indicated otherwise in a credit line to the material. If material is not included in the article's Creative Commons license and your intended use is not permitted by statutory regulation or exceeds the permitted use, you will need to obtain permission directly from the copyright holder.
To view a copy of this license, visit https://creativecommons.org/licenses/by/4.0/

Response Letter

Journal: Nature Communications

Manuscript ID: NCOMMS-26-016921-T

Title: Microchiral pinwheel arrays based on achiral molecules

Response to the Reviewers' Comments

The authors would like to express their sincere gratitude to the reviewers for their valuable feedback on our manuscript. We have carefully reviewed each comment and revised the manuscript accordingly. To enhance the readability, the reviewers' comments and the authors' responses have been color-coded in black and blue, respectively. In the revised manuscript, revised parts are highlighted in yellow.

Reviewers' Comments

Reviewer #1 (Remarks to the Author):

Yoon et al. reported that a chiral mesophase, including a racemic one, can be modulated by an electric field, and that polymerization of cyclophane molecules within this mesophase enables transfer of the mesophase chirality to the resulting polymer. They further demonstrated that the use of chiral dopants allows the formation of optically active mesophases; in such cases, micrometer-scale macroscopic chirality is induced in the polymer, and this chirality can also be transferred to an achiral luminescent dye. Ultimately, circularly polarized luminescence is generated. The generality of this chirality-transfer strategy is examined using multiple chiral dopants and luminescent dyes. The resulting structures are characterized by POM, SEM, IR, XPS, CD, and related methods. Although the results are interesting and potentially important, several issues remain unresolved and should be addressed before the manuscript can be considered for acceptance.

1. The rationale for using a cyclophane-based polymer is unclear. Why did the authors choose a cyclophane-derived polymer in this study? Since this is not a common polymer system, the authors should clarify whether there is a specific reason why this material is essential, or whether it offers particular advantages over more conventional polymers. If the authors wish to claim broad applicability of their approach, transfer to a more general polymer system should at least be discussed, if not experimentally demonstrated.

Response:

We thank the reviewer for this important comment. We agree that the rationale for using a cyclophane-derived polymer should be clarified.

PCP–OH was selected not because the present chirality-transfer concept is limited to cyclophane chemistry, but because [2.2]paracyclophane derivatives are particularly suitable for solvent-free chemical vapor deposition polymerization (CVP) on liquid-crystal mesophase templates. In our system, the chiral morphology is generated as a field-induced director configuration in the mesophase; therefore, the polymerization process must preserve this preorganized director field without disrupting the LC phase. Conventional polymers such as PE, PS, PVC, or PMMA are less suitable for this purpose because they generally show limited compatibility with liquid crystals and can cause phase separation. In addition, solution-based polymerization can disturb the mesophase through solvent swelling, capillary flow, phase separation, or changes in the local director configuration.

Response Letter

In contrast, PCP–OH is introduced as a vapor-phase precursor and polymerized under solvent-free CVP conditions, minimizing perturbation of the LC mesophase while forming PPX–OH nanofibers along the local director field. After removal of the mesophase, the resulting PPX–OH scaffold remains insoluble and mechanically stable, as confirmed by the preserved POM textures and SEM helical nanofiber morphology. Thus, PCP–OH serves as a robust model precursor for permanently fixing mesophase chirality. We have revised the manuscript to clarify this point and to avoid overstating broad polymer compatibility. We now frame extension to other vapor-deposited or LC-compatible polymer systems as a possible future direction rather than an experimentally established conclusion.

Original Manuscript Text (1):

By combining minimal dopant usage, scalable processing, and broad material compatibility, this strategy provides a general and efficient route to macroscopic chiral photonic materials built entirely from symmetric molecular precursors.

Revised Manuscript Text (1):

By combining low dopant loading, scalable processing, and solvent-free polymer fixation, this strategy provides a practical route to macroscopic chiral photonic materials based on achiral mesophase and polymer-forming components.

Original Manuscript Text (2):

The field-generated chiral templates are encoded into permanent polymer architectures using CVP of the achiral PCP–OH (4-hydroxymethyl[2.2]paracyclophane) monomer, deposited directly onto the orientationally ordered mesophase medium (Fig. 2a,b). During CVP, the monomer undergoes pyrolysis at 550–560 °C under 0.2 mbar to generate reactive p-quinodimethane diradicals, which subsequently condense and polymerize at 5 °C to produce poly(hydroxymethyl-p-xylylene) (PPX–OH) nanofibers preferentially aligned along the local director of the underlying medium (Supplementary Fig. 3)[25–28]

Revised Manuscript Text (2):

The field-generated chiral templates are encoded into permanent polymer architectures using CVP of the achiral PCP–OH (4-hydroxymethyl[2.2]paracyclophane) monomer, deposited directly onto the orientationally ordered mesophase medium (Fig. 2a,b). PCP–OH was selected because [2.2]paracyclophane derivatives enable solvent-free polymerization, allowing the preorganized director field to be fixed without solvent-induced disruption or phase separation. The resulting poly(hydroxymethyl-p-xylylene) (PPX–OH) scaffold preserves the field-induced director morphology after removal of the mesophase, providing a robust model system for mesophase-to-polymer chirality transfer. During CVP, PCP–OH undergoes pyrolysis at 550–560 °C under 0.2 mbar to generate reactive p-quinodimethane diradicals, which subsequently condense and polymerize at 5 °C to form PPX–OH nanofibers preferentially aligned along the local director of the underlying medium (Supplementary Fig. 4).[25–28]

2. The meaning of using different concentrations for different dopants is unclear. The manuscript employs different concentrations depending on the chiral dopant, but the reason for doing so is not adequately explained. A systematic study of the concentration dependence of the chiral dopant should be provided. In particular, at lower dopant concentrations, is it possible that a non-racemic mixture of right- and left-handed helices is formed? This point deserves discussion.

Response Letter

Response:

We thank the reviewer for raising this important point. We agree that the use of different dopant concentrations requires clarification.

The dopant concentrations were not chosen arbitrarily, nor were they intended for direct comparison at identical weight fractions. Instead, they were selected as the minimum concentrations that reproducibly produced single-handed pinwheel arrays under identical confinement and field conditions. Because each dopant has a different helical twisting power in MLC–6608, the concentration required to provide sufficient handedness selection differs among dopants.

To address this point, we added a concentration-dependent study for R/S-811 (Supplementary Fig. 6 (**Fig. R1–1**)). At low dopant concentrations, field-induced pinwheel arrays formed, but handedness selection was incomplete: CW and CCW domains coexisted with unequal populations, producing a mixed, non-racemic array. Upon increasing the concentration, one handedness became dominant, and uniform single-handed arrays were reproducibly obtained at 0.7 wt% R- or S-811 under the same field condition. At excessive dopant loading, fingerprint-like cholesteric textures became dominant and well-defined pinwheel structures were no longer observed. These results indicate that there is an optimal concentration window: too little dopant gives incomplete handedness selection, whereas too much dopant promotes intrinsic cholesteric ordering that competes with the field-induced pinwheel morphology.

We also clarified why R/S-5011 and CB15 were used at different concentrations. The relevant parameter is not the absolute weight fraction but the effective chiral bias imposed on the confined director field, which is reflected in the induced cholesteric pitch. R/S-5011 exhibits a stronger helical twisting power in MLC–6608 and therefore requires a lower loading than R/S-811 to produce a comparable pitch and reproducible handedness selection (Supplementary Fig. 14 (**Fig. R1–2**)). These results support our interpretation that the chiral dopant does not directly create the pinwheel defect; rather, it acts as a helicity selector that biases the handedness of the field-induced symmetry breaking.

Original Manuscript Text (1):

To impose deterministic handedness, we introduce a tiny amount (0.7 wt%) of a chiral dopant (R- or S-811) into the mesophase medium (Fig. 3a). Under the threshold field (4.5 V μm^{-1}, 10 kHz, VT), the R-811–doped system develops uniform CW spirals across the entire field of view (Fig. 3b), whereas S-811 generates uniform CCW spirals (Supplementary Fig. 5).

Revised Manuscript Text (1):

To impose deterministic handedness, we introduced a chiral dopant into the mesophase medium and examined its concentration-dependent effect on pinwheel formation under the threshold field for chiral-twist formation (4.5 V μm^{-1}, 10 kHz, (V_T)). At low R/S-811 loadings, the field-induced pinwheel arrays showed incomplete handedness selection, with minor oppositely handed domains coexisting with the dominant handedness (Supplementary Fig. 6 (**Fig. R1–1**)), indicating mixed, non-racemic arrays. At 0.7 wt%, uniform single-handed arrays were reproducibly obtained: R-811 generated homochiral CW spirals, whereas S-811 generated homochiral CCW spirals (Fig. 3b and Supplementary Fig. 6 (**Fig. R1–1**)). At higher dopant loading, fingerprint-like cholesteric textures dominated and well-defined pinwheels no longer formed, indicating that excessive loading promotes intrinsic cholesteric ordering rather than field-induced pinwheel formation handedness (Supplementary Fig. 6 (**Fig. R1–1**)). Deterministic homochiral pinwheel formation therefore requires

an optimized concentration window in which the dopant biases the handedness of the field-induced symmetry breaking without suppressing the confinement-driven morphology.

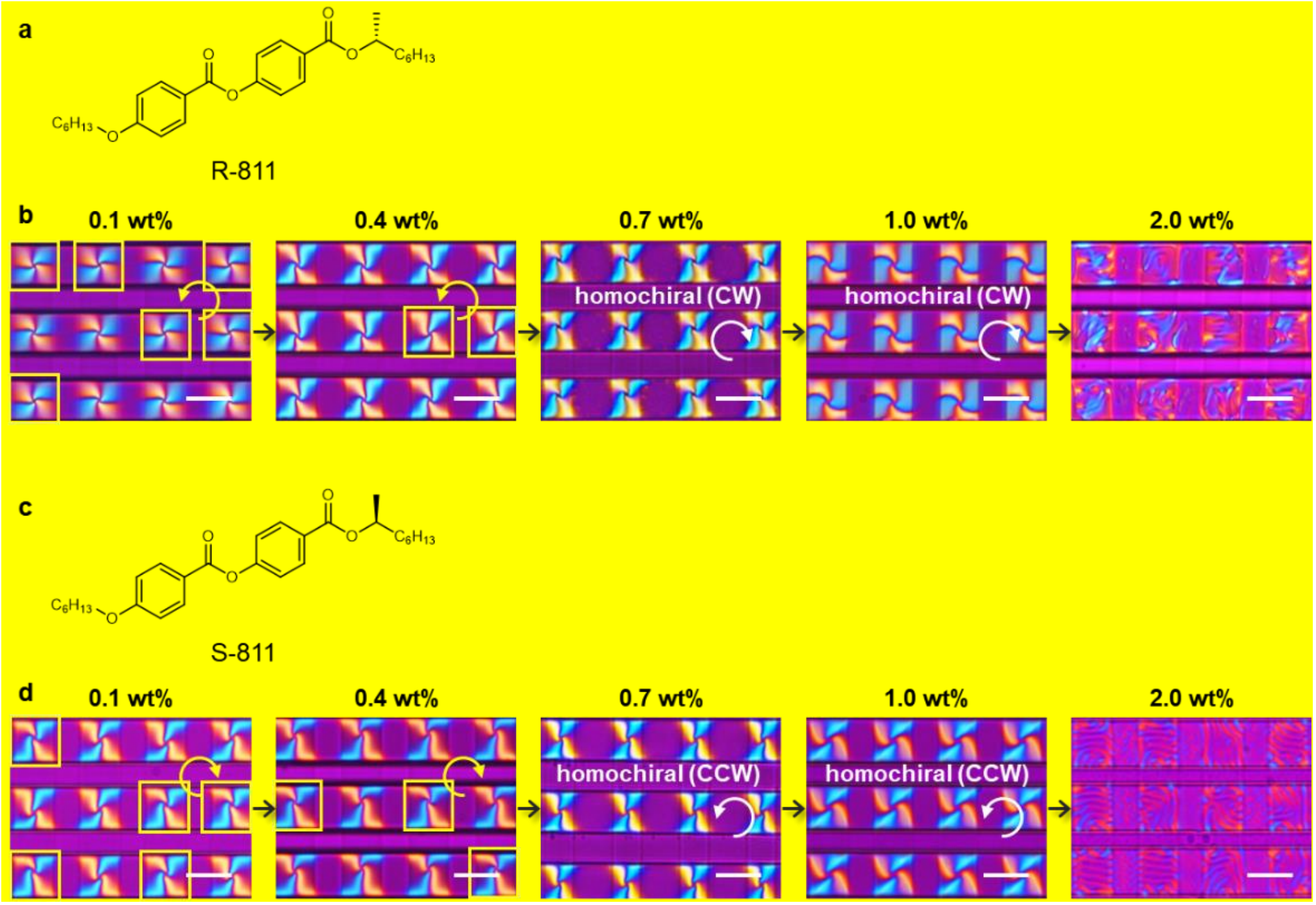

Fig. R1–1. Concentration-dependent chiral control of field-induced pinwheel arrays. a,c, Molecular structures of R-811 and S-811 chiral dopants. b,d, POM images of MLC6608 doped with different concentrations of R-811 (b) and S-811 (d) under an applied electric field of 4.5 V μm^{-1}. At 0.1 and 0.4 wt%, incomplete handedness selection is observed, with minor oppositely handed pinwheel domains highlighted by yellow boxes. At 0.7 and 1.0 wt%, the doped systems form uniform homochiral pinwheel arrays: CW for R-811 and CCW for S-811. At 2.0 wt%, fingerprint-like cholesteric textures appear, indicating that excessive dopant loading promotes intrinsic cholesteric self-assembly rather than field-induced pinwheel formation. These results identify the concentration window in which the dopant effectively biases the handedness of field-induced symmetry breaking. Scale bars, 10 μm.

Original Manuscript Text (2):

The generality and efficiency of this induction mechanism are further confirmed using chiral dopants with distinct stereochemical origins. R- or S-5011 (0.08 wt%), possessing axial chirality, and CB15 (1.1 wt%), exhibiting point chirality, both produce uniform CW and CCW homochiral domains under identical field conditions (Supplementary Figs. 7–9).

Revised Manuscript Text (2):

The applicability of this induction mechanism was further examined using chiral dopants with distinct stereochemical origins and different helical twisting powers. R/S-5011, possessing axial chirality, and CB15, exhibiting point chirality, produced uniform homochiral domains at optimized concentrations of 0.08 wt% and 1.1 wt%, respectively, under the same field condition (Supplementary Figs. 11–13). The lower concentration required for R/S-5011 reflects its stronger helical twisting power in MLC–6608. Notably, although R/S-5011 was used at a much lower concentration than R/S-811, both dopant systems produced a comparable cholesteric pitch of approximately 13 μm under their optimized conditions (Supplementary Fig. 14 (**Fig. R1–2**)), indicating that different dopant concentrations are required to provide comparable chiral bias for reproducible handedness

selection. POM, SEM, and CD analyses consistently confirm that the induced handedness follows the dopant enantiomer.

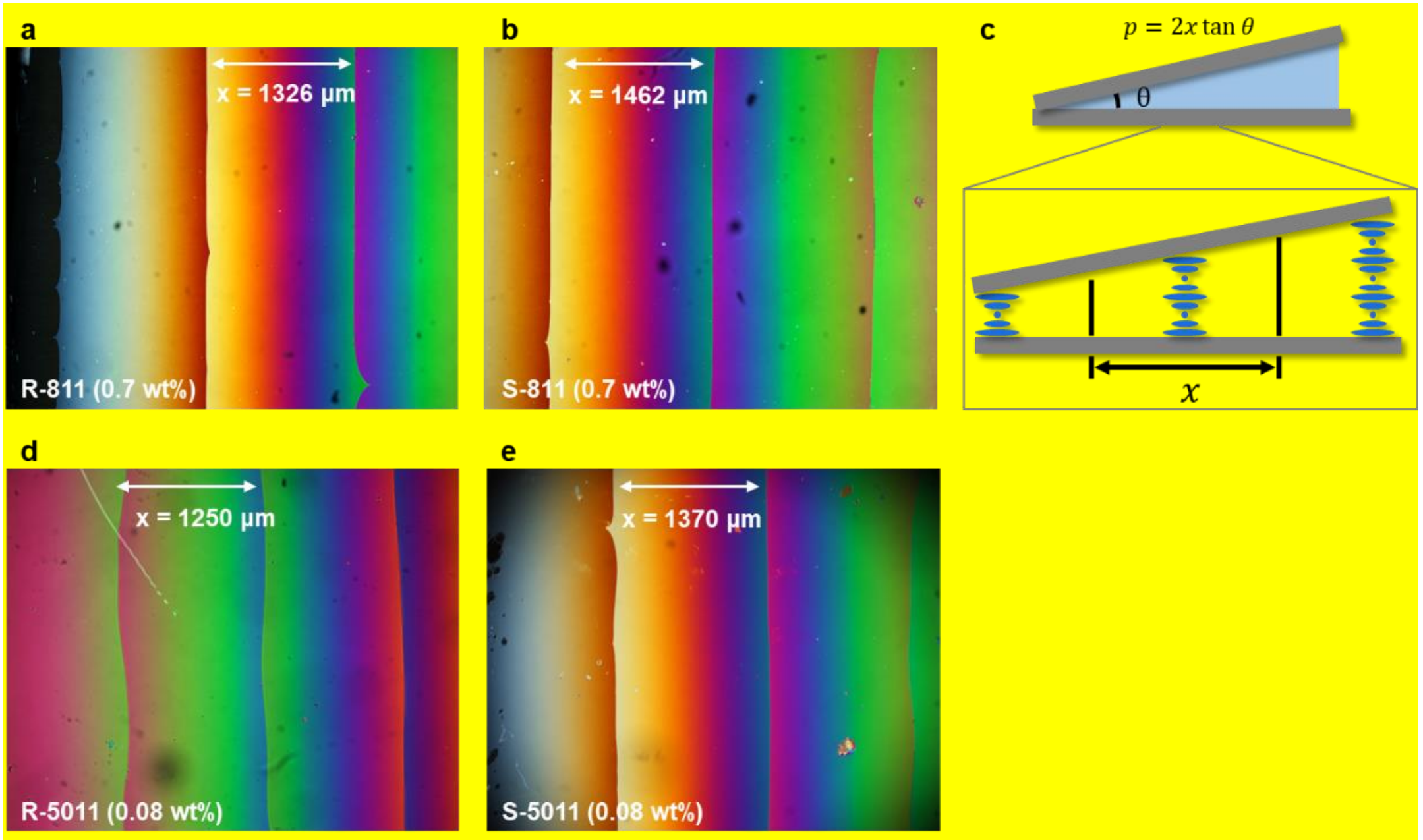

Fig. R1–2. **Cholesteric pitch measurements of R/S-811- and R/S-5011-doped MLC6608**. a,b, Polarized optical microscopy images of wedge cells prepared with MLC6608 doped with 0.7 wt% R-811 (a) and 0.7 wt% S-811 (b). The distance between adjacent Grandjean lines, x, was measured to be 1326 and 1462 μm, respectively. c, Schematic illustration of the wedge-cell method used to determine the cholesteric pitch, where the pitch was calculated using $p = 2x \tan\theta$. d,e, Polarized optical microscopy images of wedge cells prepared with MLC6608 doped with 0.08 wt% R-5011 (d) and 0.08 wt% S-5011 (e). The measured x values were 1250 and 1370 μm, respectively. Despite the much lower dopant concentration, R/S-5011 produced a cholesteric pitch comparable to that of R/S-811 at 0.7 wt%, indicating that R/S-5011 provides a stronger effective chiral bias in the MLC6608 mesophase.

3. The relationship between macroscopic helicity and molecular-level chirality remains unclear. SEM images indicate the formation of micrometer-scale helical structures, but it is not clear whether molecular- or supramolecular-level chirality is also induced within the polymer. Is the polymer essentially amorphous, or does it possess any local ordered structure? This point should be discussed in greater depth.

Response:

We thank the reviewer for raising this important point. We agree that the relationship between micrometer-scale helicity and molecular-level chirality should be clarified.

In this work, we do not claim that the achiral PPX–OH polymer becomes molecularly chiral at the covalent-chain level. Rather, the chirality of the polymer scaffold primarily arises from the supramolecular and morphological organization of PPX–OH nanofibers dictated by the field-induced LC director field. The SEM images show CW and CCW spiral morphologies at the micrometer scale, and higher-magnification SEM images reveal twisted assemblies of PPX–OH nanofibers inherited from the spiral defects. Therefore, the observed chiroptical response is attributed to the chiral arrangement of achiral PPX–OH nanofibers at the mesoscale, not to molecular chirality of the polymer backbone.

We have revised the manuscript to clarify this point. We also note that molecular-level chirality in paracyclophane-derived polymers would require the use of chiral PCP derivatives as CVP precursors, as demonstrated in previous work. In contrast, the present study intentionally uses achiral PCP–OH to demonstrate that chirality can be generated and fixed through field-guided supramolecular and morphological organization.

Original Manuscript Text:

Scanning electron microscopy (SEM) of representative domains (spots I and II) reveals well-defined CW and CCW spiral helices, and higher-magnification images confirm the twisted nanofiber arrangement inherited from the spiral defects (Fig. 2e–g). These results demonstrate that achiral molecules and an achiral PCP–OH monomer can generate a macroscopic chiral polymer architecture.

Revised Manuscript Text:

Scanning electron microscopy (SEM) of representative domains (spots I and II) reveals well-defined CW and CCW spiral helices, and higher-magnification images confirm twisted assemblies of PPX–OH nanofibers inherited from the spiral defects (Fig. 2e–g). These results demonstrate that the field-induced mesophase chirality is translated into a mesoscale chiral polymer architecture composed of achiral PPX–OH nanofibers, rather than molecular-level chirality of the polymer backbone.

4. The composition of the mesophase and thickness dependence should be clarified. The manuscript should more clearly describe what components constitute the mesophase. In addition, the dependence of CD and CPL intensity on the film thickness of both the mesophase and the polymer-containing complex should be investigated and discussed. Such analysis is necessary to distinguish intrinsic chiral optical responses from possible thickness-dependent optical artifacts.

Response:

We thank the reviewer for this important suggestion. We agree that the sample composition and thickness dependence should be clarified to distinguish genuine chiroptical responses from thickness-dependent optical artifacts.

First, we have revised the manuscript to clearly define the composition at each stage of the experiment. The mesophase template consists of the negative-dielectric-anisotropy nematic host MLC–6608, with or without a chiral dopant, confined within the VAPI microchannel array. PCP–OH is not a component of the initial mesophase; it is introduced later as a vapor-phase precursor during CVP. After CVP and subsequent washing, MLC–6608 and the chiral dopant are removed, leaving the PPX–OH polymer scaffold. For CPL measurements, the achiral luminophore is subsequently coated onto this chiral PPX–OH scaffold.

Second, we performed thickness-dependent CD measurements by varying the amount of PCP–OH precursor used during CVP from 20 to 60 mg, thereby changing the amount of deposited PPX–OH scaffold. We found that both the absolute CD intensity and (g_{abs}) values depend on the precursor amount, with the 30 mg condition giving the strongest response (Supplementary Fig. 10 (**Fig. R1–3**)). This result indicates that the magnitude of the chiroptical response is affected by the amount of deposited PPX–OH scaffold and/or nanofiber density. Importantly, despite this variation in CD magnitude, all samples retained the same CD sign and similar Cotton-effect profiles. Therefore, the thickness-dependent data show that scaffold thickness modulates the amplitude of the CD response, while the handedness-dependent sign and spectral features are preserved across different precursor amounts.

For CPL, we did not use thickness variation as the primary artifact test because the measured CPL intensity depends not only on scaffold thickness but also on dye loading, emission intensity, and optical collection geometry. Instead, we evaluated the reliability of the CPL data through dedicated optical controls, including sample rotation, racemic dye-coated scaffold controls, spectral separation between the scaffold CD band and dye emission, and analysis of possible selective reflection, as described in our response to Comment 5. These controls support that thickness-related optical artifacts are unlikely to dominate the observed CPL response and that the CPL sign and (g_{lum}) are dictated mainly by the handedness of the PPX–OH scaffold. The new CD thickness data and related discussion have been added to Supplementary Fig. 10 (**Fig. R1–3**).

Revised Manuscript Text (1):

The mesophase template consists of the negative-dielectric-anisotropy nematic host MLC–6608, with or without a chiral dopant, confined within the VAPI microchannel array and addressed by an in-plane electric field applied through the interdigitated ITO electrodes. PCP–OH is introduced only during the subsequent CVP step as a vapor-phase precursor, and MLC–6608 and the dopant are removed after polymerization to leave the PPX–OH scaffold.

Revised Manuscript Text (2):

We further varied the PCP–OH precursor amount during CVP to control the polymer scaffold thickness (Supplementary Fig. 10 (Fig. R1–3)). The absolute CD intensity and (g_{abs}) values varied with precursor amount, with the 30 mg condition giving the strongest response, indicating that the chiroptical magnitude is influenced by scaffold thickness and/or nanofiber density. Nevertheless, all samples retained the same CD sign and similar Cotton-effect profiles, supporting that the handedness-dependent CD response originates from the chiral nanofiber architecture, while its magnitude is modulated by the deposited scaffold amount.

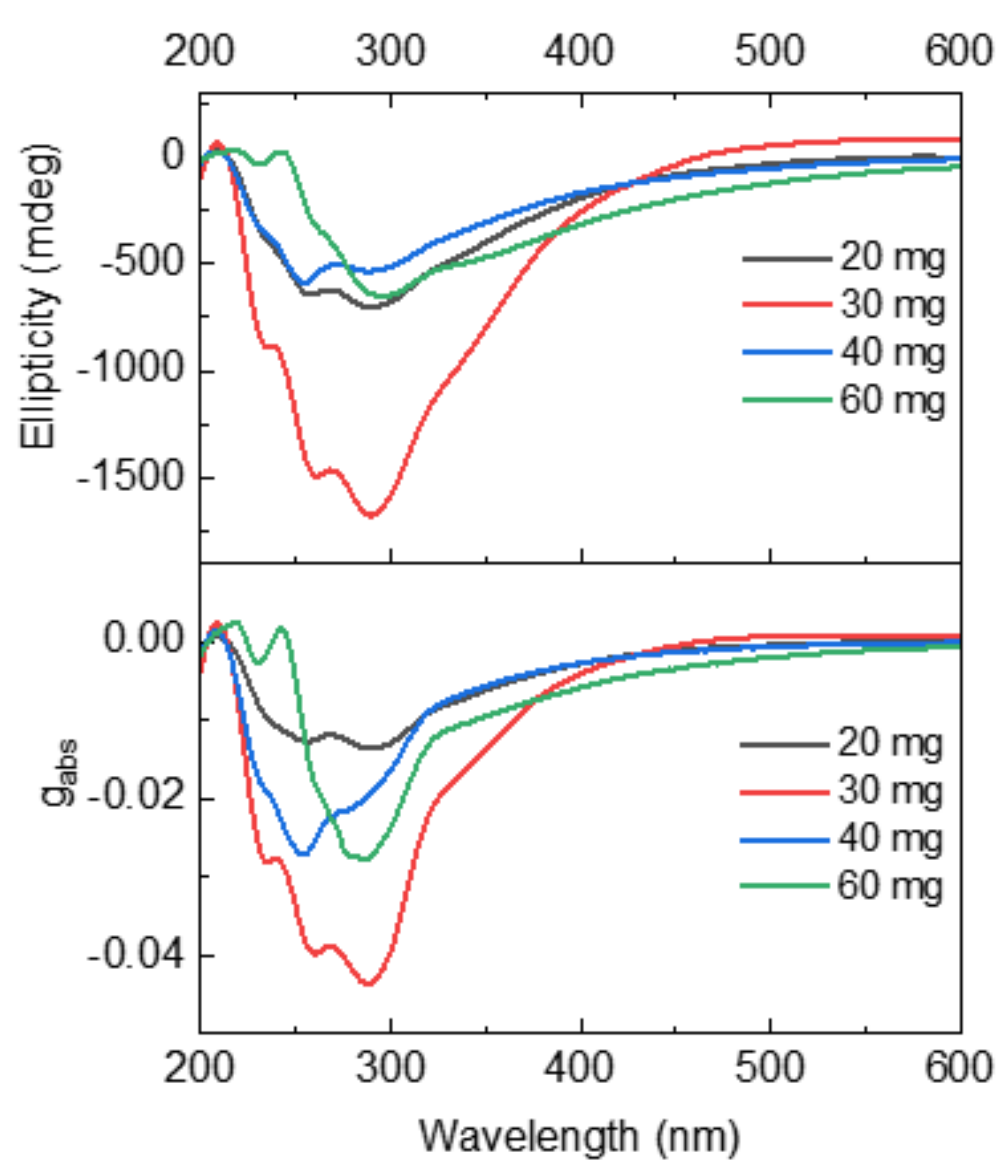

Fig. R1–3. PCP–OH precursor amount-dependent CD response of S-811-templated PPX–OH scaffolds. CD spectra and corresponding (g_{abs}) spectra of S-811-templated PPX–OH scaffolds prepared by CVP using different PCP–OH precursor amounts. The CD intensity and (g_{abs}) values vary with precursor amount, with the 30 mg condition showing the strongest response. This indicates that the chiroptical magnitude is affected by the amount of deposited PPX–OH scaffold and/or nanofiber density. Despite this variation, all samples retain the

same negative Cotton effect and similar spectral profiles, supporting that the handedness-dependent CD response is preserved across different scaffold thicknesses.

5. Although CD and CPL are reported in the solid state, there is essentially no discussion of anisotropy-related artifacts. For solid-state CD measurements, the possible contributions of linear dichroism and linear birefringence should be carefully examined. For CPL measurements, possible contamination from CD as well as the effects of selective scattering and selective reflection should also be thoroughly evaluated. At present, the reliability of these optical data is not fully guaranteed.

Response:

We thank the reviewer for raising this important point. We agree that solid-state CD and CPL measurements can be affected by anisotropy-related artifacts, including linear dichroism (LD), linear birefringence (LB), sample-orientation effects, scattering, selective reflection, and polarization leakage. We therefore performed additional control experiments to evaluate the reliability of the chiroptical data.

For CD measurements, we recorded spectra while rotating the sample about the beam axis at 0°, 45°, 90°, and 135° (Supplementary Fig. S9 (**Fig. R1–4**)). If the signal were dominated by macroscopic LD/LB artifacts, substantial changes in spectral shape or sign would be expected upon rotation. In contrast, the handedness-dependent CD sign and spectral shape were preserved at all rotation angles, with only minor variations in amplitude. We also performed front- and back-side measurements by flipping the sample. Although small intensity differences can arise from changes in optical path and substrate/air-interface geometry, the main spectral features and handedness-dependent sign were retained. In addition, the undoped racemic scaffold showed a nearly flat CD baseline. Together with the thickness-dependent CD measurements described in Comment 4, these controls indicate that the CD magnitude is influenced by scaffold amount, but the handedness-dependent sign and spectral profile are not dominated by LD/LB, sample orientation, or substrate-related artifacts.

For CPL measurements, we recorded spectra during full in-plane sample rotation in 45° steps from 0° to 315° (Supplementary Fig. 15 (**Fig. R1–5**)). For the R-811-templated scaffold, the CPL signal remained positive at all rotation angles, whereas the S-811-templated scaffold retained negative CPL at all angles. The overall spectral shape was preserved upon rotation, and the g_{lum} values showed only moderate angular variation without sign reversal. These results indicate that the CPL response is not dominated by sample-orientation artifacts. In addition, the dye-coated racemic scaffold showed negligible CPL, whereas dye-coated homochiral scaffolds produced opposite CPL signs depending on the scaffold handedness. Together, these controls support that the CPL originates primarily from chirality transfer from the homochiral PPX–OH scaffold rather than from the achiral dye film or instrumental polarization artifacts.

We also considered possible CD contamination, selective scattering, and selective reflection. The main CD response of the PPX–OH scaffold appears in the ultraviolet region, whereas the green luminophore emits in the visible region around 540–550 nm. This spectral separation reduces the likelihood of direct CD-based differential absorption contributing to the CPL signal at the emission wavelength. In addition, based on the measured cholesteric pitch of approximately 13 μm and an estimated refractive index of ~1.5, the corresponding selective-reflection wavelength would lie in the mid-infrared region, far from the visible emission window. Therefore, selective reflection is unlikely to be the primary origin of the observed CPL. Together with the racemic dye-coated control and rotation-invariant CPL response, these results support that the observed CPL originates from

the handedness of the chiral PPX–OH scaffold rather than from anisotropy-related optical artifacts. We have revised the manuscript to present these conclusions in a more cautious manner.

Revised Manuscript Text (1):

We next performed control experiments to evaluate possible solid-state optical artifacts. CD spectra measured at sample-rotation angles of 0°, 45°, 90°, and 135° and in front- and back-side geometries retained the same handedness-dependent sign and spectral shape (Supplementary Fig. 9 (**Fig. R1–4**)), indicating that the response is not dominated by linear dichroism, linear birefringence, or sample-orientation effects.

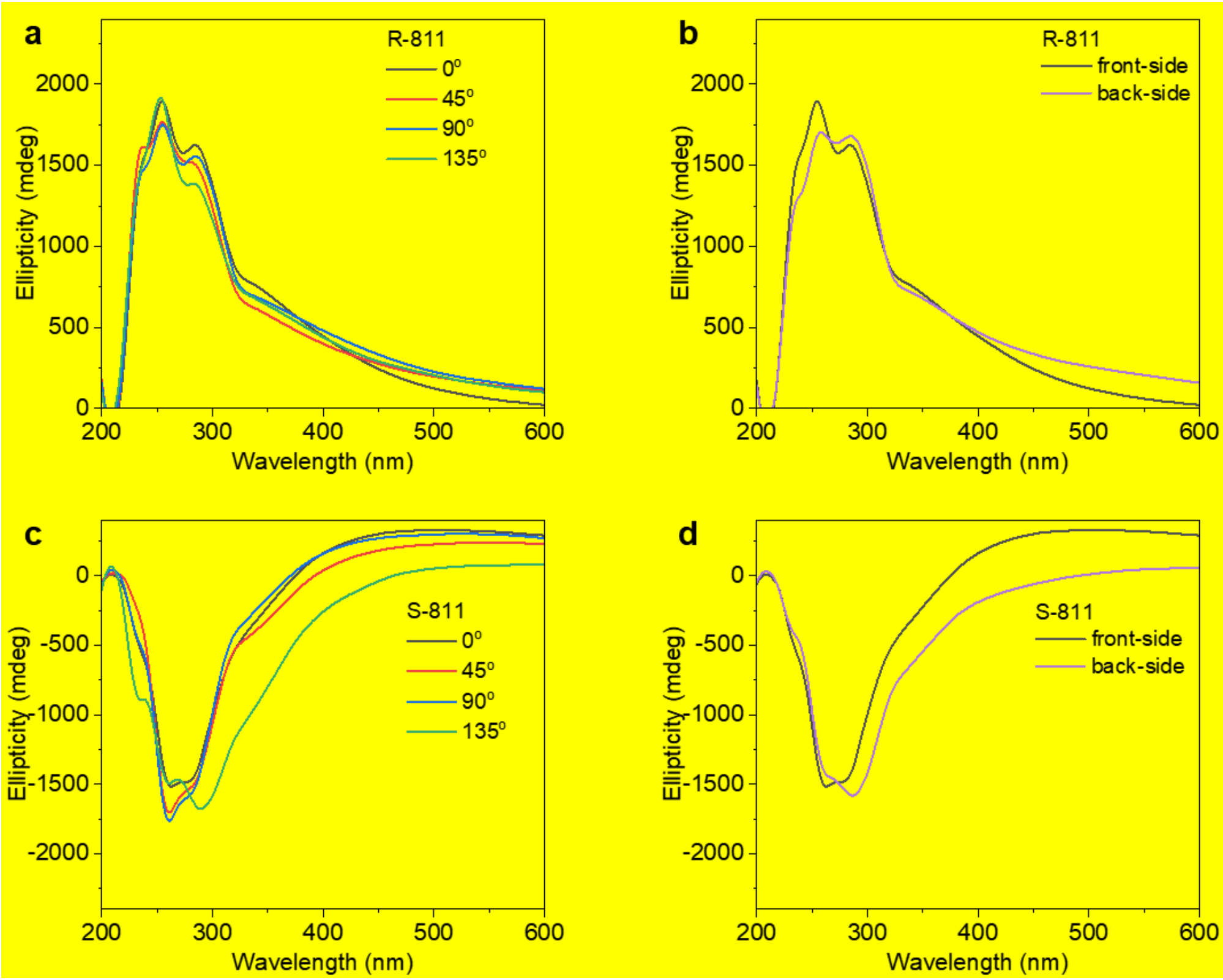

Fig. R1–4. Orientation-dependent CD control measurements of R-811- and S-811-templated PPX–OH scaffolds. (a,c) CD spectra of the R-811- and S-811-templated scaffolds measured after in-plane sample rotation at 0°, 45°, 90°, and 135°. The handedness-dependent CD sign and overall spectral shape are preserved at all rotation angles, indicating that the observed CD response is not dominated by sample-orientation-dependent linear dichroism or linear birefringence artifacts. (b,d) Front- and back-side CD measurements of the R-811- and S-811-templated scaffolds. The spectra retain the same handedness-dependent sign and similar spectral features after flipping the sample, supporting that the CD response primarily originates from the chiral nanofiber architecture rather than from substrate- or measurement-geometry-induced artifacts.

Revised Manuscript Text (2):

To evaluate possible sample-orientation artifacts in the CPL measurements, we recorded CPL spectra during full in-plane sample rotation in 45° steps from 0° to 315° (Supplementary Fig. 15 (Fig. R1–5)). The R-811-templated scaffold retained positive CPL at all rotation angles, whereas the S-811-templated scaffold retained negative CPL. Although the ellipticity and (g_{lum}) values showed moderate angular variation, the CPL sign and overall spectral shape were preserved for each handed scaffold, supporting that the observed CPL is not dominated by sample-orientation artifacts. In addition, the scaffold CD band lies mainly in the ultraviolet region,

whereas the dye emission occurs around 540–550 nm, and the estimated selective-reflection wavelength is far outside the visible emission region. These controls support that the CPL arises from chirality transfer from the homochiral PPX–OH scaffold to the achiral luminophore, rather than being dominated by sample orientation, CD leakage, or selective-reflection effects.

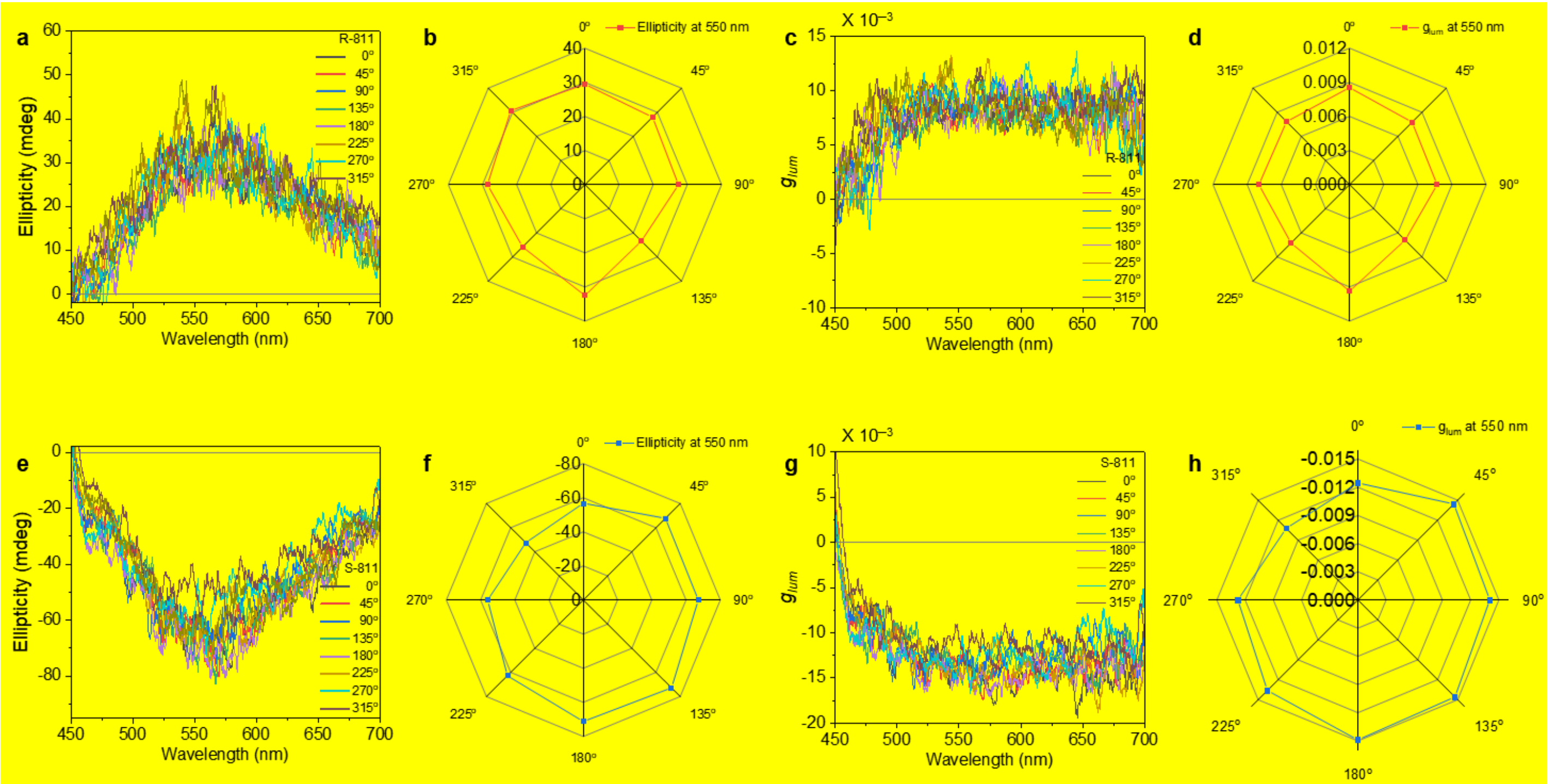

Fig. R1–5. Angle-dependent CPL control measurements of dye-coated R-811- and S-811-templated PPX–OH scaffolds. (a,e) CPL spectra of dye-coated PPX–OH scaffolds templated by (a) R-811 and (e) S-811, measured during in-plane sample rotation from 0° to 315° in 45° steps. The R-811-templated scaffold retains positive CPL at all rotation angles, whereas the S-811-templated scaffold retains negative CPL. (b,f) Polar plots of CPL ellipticity at 550 nm as a function of sample-rotation angle. (c,g) Corresponding g_{lum} spectra measured at each rotation angle. (d,h) Polar plots of g_{lum} at 550 nm. Although the ellipticity and g_{lum} values show moderate angular variation, the CPL sign and overall spectral shape are preserved for each handed scaffold, supporting that the observed CPL response is not dominated by sample-orientation artifacts.

6. For samples that are expected to be enantiomeric counterparts, the CD spectra should normally exhibit clear mirror-image relationships. The fact that the spectra in Fig. 2h do not do so suggests that factors such as differences in film thickness, sample orientation, scattering, or incomplete enantiomeric inversion may be involved. The authors should explain this point.

Response:

We agree with the reviewer that the local CD spectra in Fig. 2h should not be interpreted as ideal mirror-image spectra. The spectra in Fig. 2h were obtained from two different microscopic domains within a racemic polymer film using a micro-focused CD setup, rather than from a rigorously prepared pair of enantiomeric samples. Therefore, they reflect local chiroptical responses from selected CW and CCW regions rather than ensemble-averaged CD spectra of macroscopically uniform enantiomeric films.

Accordingly, local differences in polymer thickness, nanofiber density, director tilt, optical path length, sample orientation, and scattering can affect the absolute CD intensity and spectral shape. The key point of Fig. 2h is that

CW and CCW domains exhibit opposite-sign CD responses, consistent with the handedness observed by POM and SEM. However, because the two measured spots are local domains with different microenvironments, perfect mirror-image symmetry is not expected. We have revised the manuscript to avoid overinterpreting these spectra as ideal enantiomeric counterparts.

Original Manuscript Text:

Focused circular dichroism (CD) spectroscopy (Fig. 2h) shows opposite-sign signals for CW and CCW regions, while spatial CD mapping (Fig. 2i,j) reveals a racemic mixture of chiral domains, illustrating a macroscopic chiral polymer framework through confinement and the applied field.

Revised Manuscript Text:

Focused circular dichroism (CD) spectroscopy (Fig. 2h) shows opposite-sign Cotton effects from representative CW and CCW regions. Because these spectra were collected from different microscopic domains in a racemic film, local variations in polymer thickness, fiber density, orientation, and scattering can produce deviations from ideal mirror-image symmetry. Spatial CD mapping (Fig. 2i,j) reveals a racemic distribution of chiral domains, illustrating the formation of a macroscopic chiral polymer framework through confinement and the applied field.

7. The chemical structures of the dopants should be shown in the manuscript.

Response:

We thank the reviewer for this suggestion. We have revised Fig. 3a to include the chemical structure of the chiral dopants.

Response Letter

Reviewer #2 (Remarks to the Author):

The manuscript reports preparation and characterization of chiral structure from achiral building blocks, based on electric-field generated array of defects in director field. Originally racemic array can be turned into homochiral state by using chiral dopant, which breaks degeneracy of enantiomeric structures. Authors used a nematic LC confined in regular system of microchannels prepared from VAPI, which was exposed to in-plane electric field generated by comb-like electrodes. Under such condition, on the electrodes the pinwheel defects were generated, which can be used as templates to generate stable chiral arrangement of polymer fibers by CVP. Authors convincingly demonstrated possibility of obtaining chiral structures, e.g for generating of circularly polarized luminescence from achiral dye. In general, the subject is interesting, and reported results might deserve publication in NatComm. However, few points should be clarified/presented in more clear way:

1. Mechanism of chiral defect creation is not clearly described, why pinwheel arrangement is favored over simple point -1 defects? The equations provided in SM are very general and to not provide the mechanism.

Response:

We thank the reviewer for this important comment. We agree that the original manuscript did not sufficiently explain why the field-induced defect relaxes into a pinwheel-like arrangement rather than a simple −1 point defect. We have therefore revised the main text and added a new numerical director-field simulation in Supplementary Fig. S3 (**Fig. R2–1**) to provide a clearer physical description of the three-dimensional director-field mechanism.

The numerical simulation was performed using the experimental device geometry and the supplier-provided dielectric and elastic parameters of MLC–6608. Under the imposed homeotropic anchoring and in-plane electric field, the director field relaxes into a pinwheel-like splay–bend–twist configuration, rather than a −1 point defect. This supports our interpretation that the observed pinwheel is not an ad hoc assignment, but a three-dimensional director configuration selected by the combined boundary and field constraints.

- **Competing boundary and field effects:** The VAPI boundaries and molecule–air interface impose predominantly homeotropic alignment, whereas the in-plane electric field induces director reorientation because MLC–6608 has negative dielectric anisotropy.
- **Localized instability near electrode gaps:** The nonuniform electric field near the electrode gaps creates regions where the balance between electric torque and anchoring elasticity becomes unstable, leading to defect nucleation.
- **Three-dimensional director relaxation:** Within the VAPI microchannels, the director must satisfy the anchoring conditions at the bottom surface, sidewalls, and molecule–air interface. A −1 point defect cannot efficiently accommodate these constraints, so the director relaxes through a combined splay–bend–twist deformation.
- **Degenerate handedness in the achiral host:** The resulting twist–spiral configuration can adopt either CW or CCW handedness with equal probability in the achiral host, producing a racemic distribution of pinwheel domains.

Thus, the pinwheel morphology is favored because it provides a three-dimensional relaxation pathway for the confined director field under in-plane electric-field torque. In addition, our group has previously reported a prior study on similar type of defect generation[1]. We have revised the manuscript accordingly and added the following text.

Response Letter

1. Kim, D. S., Čopar, S., Tkalec, U. & Yoon, D. K. Mosaics of topological defects in micropatterned liquid crystal textures. Sci. Adv. 4, 8064–8087 (2018).

Revised Manuscript Text:

This pinwheel texture is better described as a three-dimensional director distortion than as a simple two-dimensional −1 point defect (Supplementary Fig. 3 (**Fig. R2–1)**). It arises from the combined effects of vertical anchoring, sidewall confinement within the VAPI microchannels, and the in-plane electric field. Because MLC–6608 has negative dielectric anisotropy (Supplementary Table 1), the director tends to reorient perpendicular to the applied field while simultaneously satisfying the anchoring conditions imposed by the VAPI boundaries and the molecule–air interface. This competition promotes coupled splay, bend, and twist deformations, yielding the pinwheel-like twist–spiral arrangement.

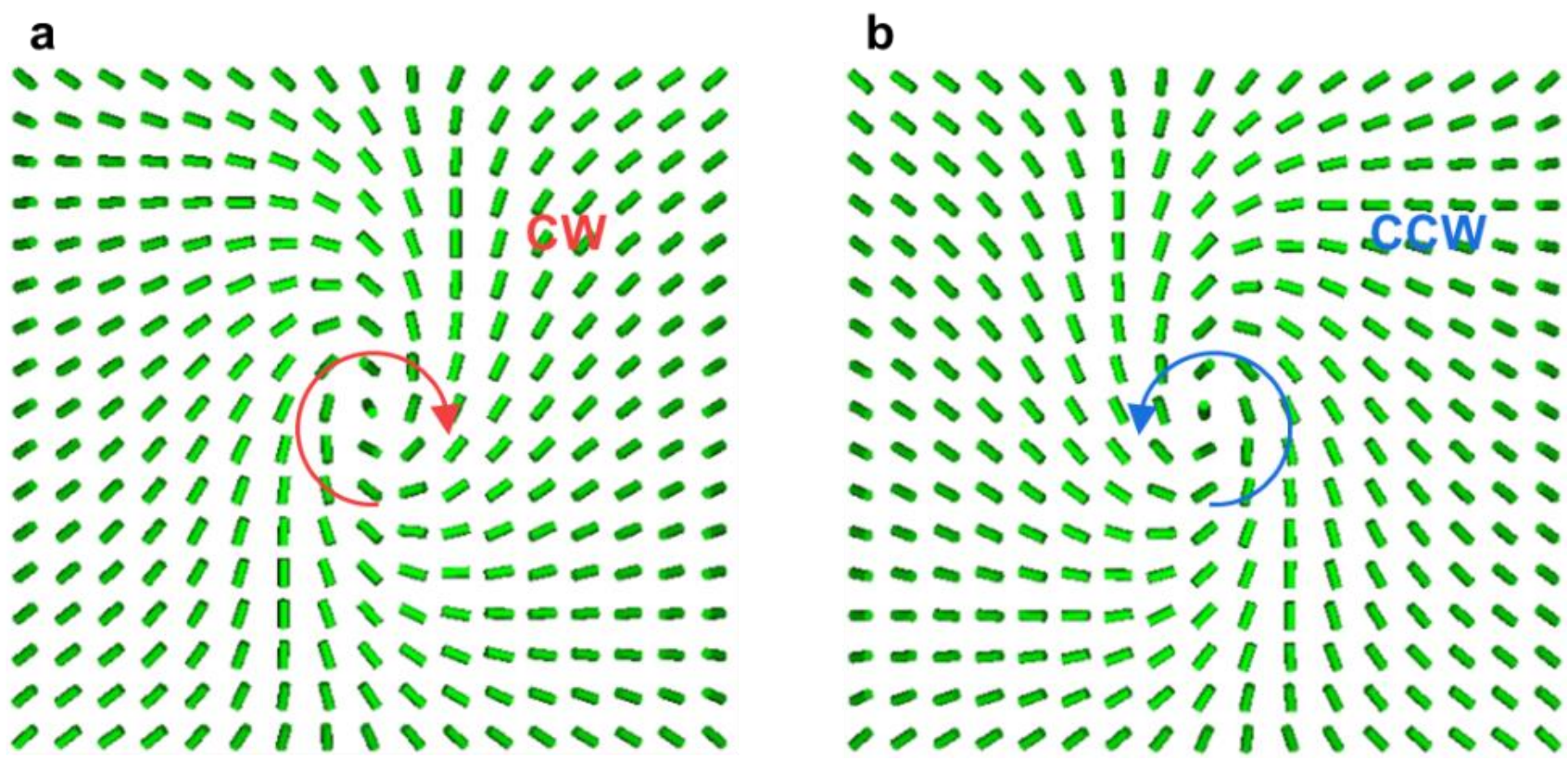

Fig. R2–1. Numerical simulation of director-field configurations for pinwheel domains. Simulated in-plane director-field maps showing representative (a) clockwise (CW) and (b) counterclockwise (CCW) pinwheel configurations generated under the confined geometry and applied in-plane electric field. Green rods indicate the local orientation of the LC director. The simulated director field relaxes into a twist–spiral arrangement rather than a simple two-dimensional −1 point defect, supporting that the experimentally observed pinwheel texture originates from a three-dimensional splay–bend–twist distortion of the confined MLC–6608 mesophase. Red and blue arrows indicate the rotational sense of the CW and CCW domains, respectively.

2. I would suggest to give more detailed scheme of 3D director field in the defect region

Response:

We thank the reviewer for this helpful suggestion. As suggested, we have added a more detailed three-dimensional director-field simulation of the defect region as Supplementary Fig. S3. As discussed in our response to Comment 1, this figure shows how vertical anchoring, microchannel confinement, and the in-plane electric field collectively generate the pinwheel-like twist–spiral configuration. The corresponding explanation has also been incorporated into the revised main text.

3. Figure S1b suggest that the used LC has a negative dielectric anisotropy -is it true? The data regarding dielectric anisotropy and elastic constants of the material should be provided

Response:

Response Letter

We thank the reviewer for pointing this out. Yes, MLC–6608 has negative dielectric anisotropy. We have clarified this point in the revised manuscript and incorporated it into the mechanistic discussion of the field-induced pinwheel formation, as described in our response to Comment 1.

Specifically, the negative dielectric anisotropy of MLC–6608 causes the director to preferentially reorient perpendicular to the applied in-plane electric field. This field-induced reorientation competes with the homeotropic anchoring imposed by the VAPI boundaries and the molecule–air interface, contributing to the three-dimensional splay–bend–twist director distortion that gives rise to the pinwheel-like texture.

We have also revised the Supplementary Information to provide the dielectric and elastic parameters of MLC–6608. The supplier-provided dielectric constants are ($\varepsilon_{\parallel}$=3.6 and $\varepsilon_{\perp}$=7.8), giving a negative dielectric anisotropy of $\Delta\varepsilon=\varepsilon_{\parallel}-\varepsilon_{\perp}=-4.2$). The elastic constants used for the director-field simulation are K_1 = 16.7 pN, K_2 = 7.3 pN, and K_3 = 18.1 pN. The relatively small twist elastic constant (K_2) supports the involvement of twist deformation in the observed splay–bend–twist director configuration. These parameters are summarized in Supplementary Table 1 (**Table R2–1**).

Table R2–1. Dielectric and elastic parameters of MLC–6608.

**Parameter**	**Symbol**	**Value**
Parallel dielectric constant	$\varepsilon_{\parallel}$	3.6
Perpendicular dielectric constant	$\varepsilon_{\perp}$	7.8
Dielectric anisotropy	$\Delta\varepsilon=\varepsilon_{\parallel}-\varepsilon_{\perp}$	-4.2
Splay elastic constant	K_1	16.7 pN
Twist elastic constant	K_2	7.3 pN
Bend elastic constant	K_3	18.1 pN

MLC–6608 is a nematic LC with negative dielectric anisotropy. According to the supplier-provided material parameters, $\varepsilon_{\parallel}$=3.6, $\varepsilon_{\perp}$=7.8, giving a negative dielectric anisotropy of $\Delta\varepsilon=\varepsilon_{\parallel}-\varepsilon_{\perp}=-4.2$. Therefore, under an applied in-plane electric field, the director tends to reorient perpendicular to the electric-field direction. This field-induced reorientation competes with the homeotropic anchoring imposed by the VAPI boundaries and the molecule–air interface, contributing to the director distortion described in the main text. The elastic constants used in the director-field simulation are (K_1 = 16.7) pN, (K_2 = 7.3) pN, and (K_3 = 18.1) pN, corresponding to splay, twist, and bend deformations, respectively. The comparatively small (K_2) value suggests that twist deformation is more accessible than splay or bend deformation, which may facilitate the twist component of the observed pinwheel-like splay–bend–twist configuration.

4. Direction o coordination axis system presented in Figure S1 do not gree with description of walls and electrodes direction (see line 74,75)

Response:

We thank the reviewer for carefully pointing out this inconsistency. We have revised the coordinate-axis labels in Supplementary Fig. S1 so that the electrode direction, electric-field direction, and VAPI wall/channel geometry are consistent with the description in the main text.

5. Concentration of used chiral dopany (~2%) should not be refered to as ultralow, its rather standard for commercial mixtures.

Response:

We agree with the reviewer that the term “ultralow” may be misleading, particularly because chiral dopant concentrations around 1–2 wt% are common in commercial cholesteric mixtures. We have therefore removed “ultralow” throughout the manuscript and replaced it with more neutral descriptions such as “low dopant loading” or “optimized dopant concentration.”

We also clarified that the concentrations used in this study were not chosen arbitrarily or for comparison at identical mass fractions. Instead, they were selected as the minimum concentrations that reproducibly produced single-handed pinwheel arrays under identical confinement and field conditions. Because each dopant has a different helical twisting power in MLC–6608, the concentration required to provide sufficient handedness selection differs among dopants.

To address this point, we added a concentration-dependent study for R/S-811 (Supplementary Fig. 6 (**Fig. R2–2**)). At low dopant concentrations, field-induced pinwheel arrays formed, but handedness selection was incomplete: CW and CCW domains coexisted with unequal populations, producing a mixed, non-racemic array. Upon increasing the concentration, one handedness became dominant, and uniform single-handed arrays were reproducibly obtained at 0.7 wt% R- or S-811 under the same field condition. At excessive dopant loading, fingerprint-like cholesteric textures became dominant and well-defined pinwheel structures were no longer observed. These results indicate that there is an optimal concentration window: too little dopant gives incomplete handedness selection, whereas too much dopant promotes intrinsic cholesteric ordering that competes with the field-induced pinwheel morphology.

We also clarified why R/S-5011 and CB15 were used at different concentrations. The relevant parameter is not the absolute weight fraction but the effective chiral bias imposed on the confined director field, which is reflected in the induced cholesteric pitch. R/S-5011 exhibits a stronger helical twisting power in MLC–6608 and therefore requires a lower loading than R/S-811 to produce a comparable pitch and reproducible handedness selection (Supplementary Fig. 14 (**Fig. R2–3**)). These results support our interpretation that the chiral dopant does not directly create the pinwheel defect; rather, it acts as a helicity selector that biases the handedness of the field-induced symmetry breaking.

Original Manuscript Text (1):

To impose deterministic handedness, we introduce a tiny amount (0.7 wt%) of a chiral dopant (R- or S-811) into the mesophase medium (Fig. 3a). Under the threshold field (4.5 V μm^{-1}, 10 kHz, VT), the R-811–doped system develops uniform CW spirals across the entire field of view (Fig. 3b), whereas S-811 generates uniform CCW spirals (Supplementary Fig. 5).

Revised Manuscript Text (1):

To impose deterministic handedness, we introduced a chiral dopant into the mesophase medium and examined its concentration-dependent effect on pinwheel formation under the threshold field for chiral-twist formation (4.5 V μm^{-1}, 10 kHz, (V_T)). At low R/S-811 loadings, the field-induced pinwheel arrays showed incomplete handedness selection, with minor oppositely handed domains coexisting with the dominant handedness (Supplementary Fig. 6 (**Fig. R2–2**)), indicating mixed, non-racemic arrays. At 0.7 wt%, uniform

single-handed arrays were reproducibly obtained: R-811 generated homochiral CW spirals, whereas S-811 generated homochiral CCW spirals (Fig. 3b and Supplementary Fig. 6 (**Fig. R2–2**)). At higher dopant loading, fingerprint-like cholesteric textures dominated and well-defined pinwheels no longer formed, indicating that excessive loading promotes intrinsic cholesteric ordering rather than field-induced pinwheel formation handedness (Supplementary Fig. 6 (**Fig. R2–2**)). Deterministic homochiral pinwheel formation therefore requires an optimized concentration window in which the dopant biases the handedness of the field-induced symmetry breaking without suppressing the confinement-driven morphology.

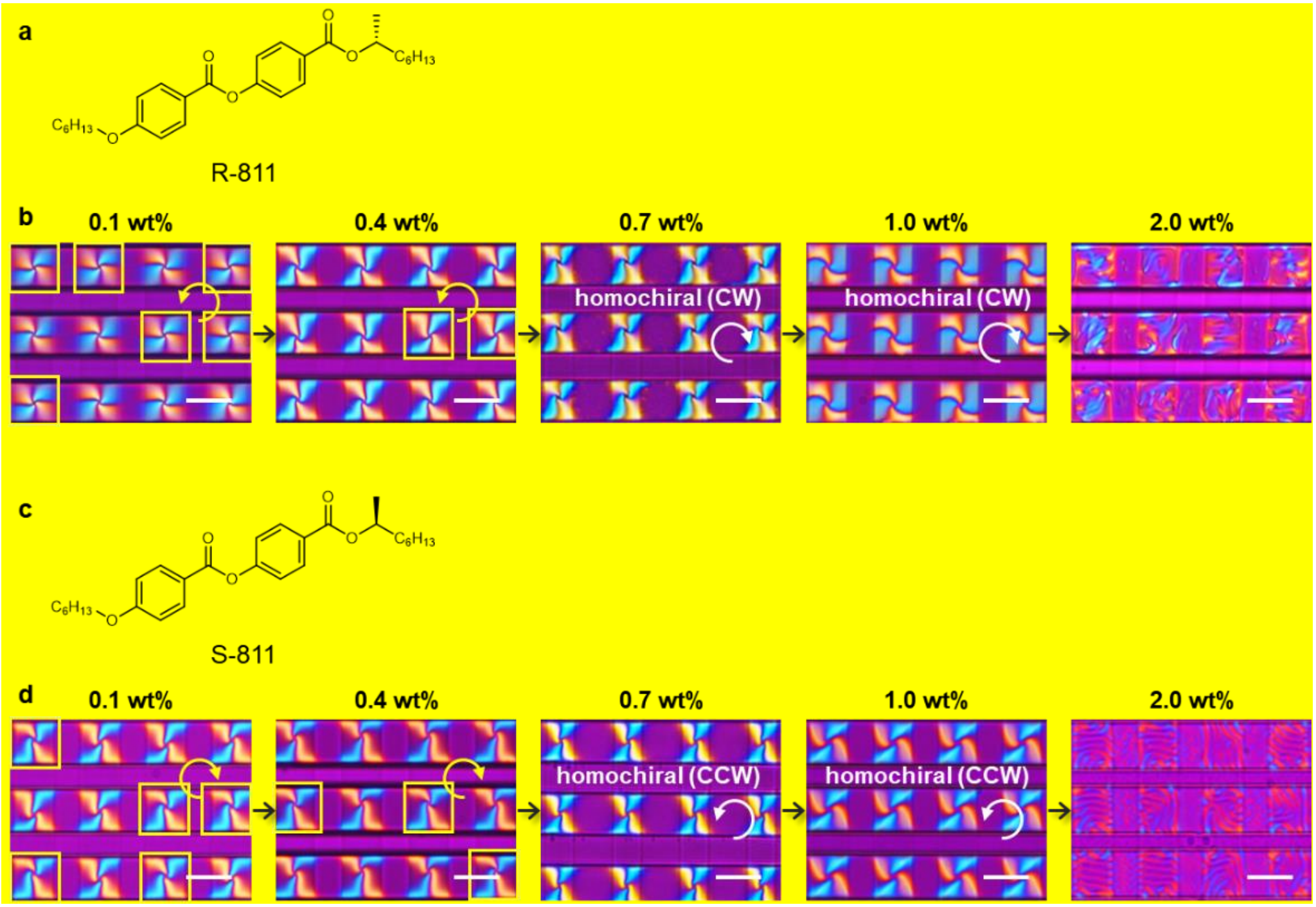

Fig. R2–2. Concentration-dependent chiral control of field-induced pinwheel arrays. a,c, Molecular structures of R-811 and S-811 chiral dopants. b,d, POM images of MLC6608 doped with different concentrations of R-811 (b) and S-811 (d) under an applied electric field of 4.5 V μm-1. At 0.1 and 0.4 wt%, incomplete handedness selection is observed, with minor oppositely handed pinwheel domains highlighted by yellow boxes. At 0.7 and 1.0 wt%, the doped systems form uniform homochiral pinwheel arrays: CW for R-811 and CCW for S-811. At 2.0 wt%, fingerprint-like cholesteric textures appear, indicating that excessive dopant loading promotes intrinsic cholesteric self-assembly rather than field-induced pinwheel formation. These results identify the concentration window in which the dopant effectively biases the handedness of field-induced symmetry breaking. Scale bars, 10 μm.

Original Manuscript Text (2):

The generality and efficiency of this induction mechanism are further confirmed using chiral dopants with distinct stereochemical origins. R- or S-5011 (0.08 wt%), possessing axial chirality, and CB15 (1.1 wt%), exhibiting point chirality, both produce uniform CW and CCW homochiral domains under identical field conditions (Supplementary Figs. 7–9).

Revised Manuscript Text (2):

The applicability of this induction mechanism was further examined using chiral dopants with distinct stereochemical origins and different helical twisting powers. R/S-5011, possessing axial chirality, and CB15, exhibiting point chirality, produced uniform homochiral domains at optimized concentrations of 0.08 wt% and 1.1

wt%, respectively, under the same field condition (Supplementary Figs. 11–13). The lower concentration required for R/S-5011 reflects its stronger helical twisting power in MLC–6608. Notably, although R/S-5011 was used at a much lower concentration than R/S-811, both dopant systems produced a comparable cholesteric pitch of approximately 13 μm under their optimized conditions (Supplementary Fig. 14 (**Fig. R2–3**)), indicating that different dopant concentrations are required to provide comparable chiral bias for reproducible handedness selection.

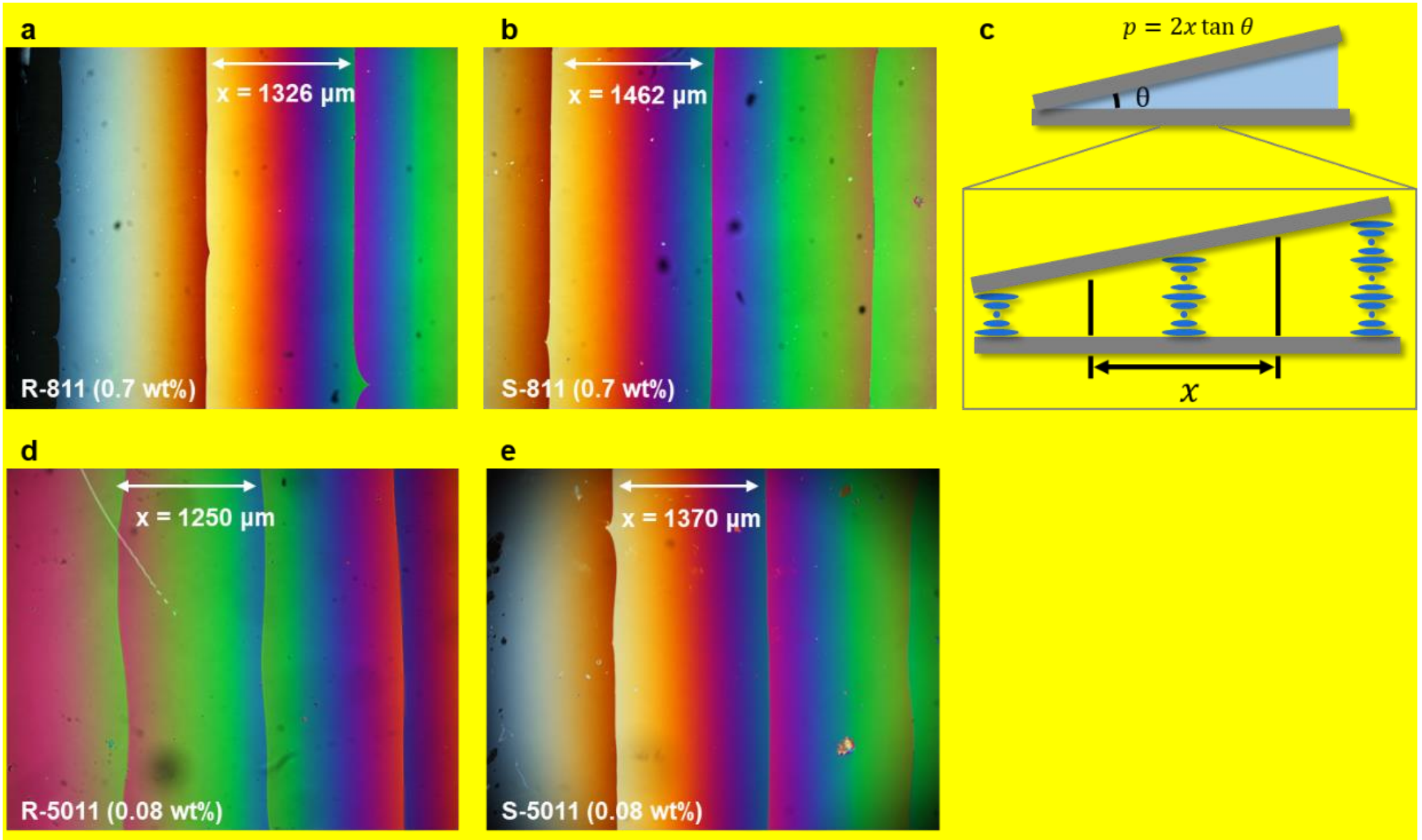

Fig. R2–3. **Cholesteric pitch measurements of R/S-811- and R/S-5011-doped MLC6608**. a,b, Polarized optical microscopy images of wedge cells prepared with MLC6608 doped with 0.7 wt% R-811 (a) and 0.7 wt% S-811 (b). The distance between adjacent Grandjean lines, x, was measured to be 1326 and 1462 μm, respectively. c, Schematic illustration of the wedge-cell method used to determine the cholesteric pitch, where the pitch was calculated using $p = 2x \tan\theta$. d,e, Polarized optical microscopy images of wedge cells prepared with MLC6608 doped with 0.08 wt% R-5011 (d) and 0.08 wt% S-5011 (e). The measured x values were 1250 and 1370 μm, respectively. Despite the much lower dopant concentration, R/S-5011 produced a cholesteric pitch comparable to that of R/S-811 at 0.7 wt%, indicating that R/S-5011 provides a stronger effective chiral bias in the MLC6608 mesophase.
